# Induction and Rapid Orientation of Agency Nursing Staff in the Hospital Setting: A Systematic Synthesis of Qualitative Studies

**DOI:** 10.1111/jan.16840

**Published:** 2025-02-27

**Authors:** Josephine Telschow, Almuth Berg

**Affiliations:** ^1^ Institute of Health and Nursing Science, Medical Faculty Martin Luther University Halle‐Wittenberg Halle Germany; ^2^ Department of Nursing Science and Practice Development Charité University Medicine Berlin Berlin Germany

**Keywords:** agency nursing, employment, hospitals, induction, inservice training, nursing staff, orientation, qualitative research, staff attitude, temporary staff

## Abstract

**Aim:**

To aggregate the experiences of temporary nursing staff in the hospital setting related to their induction and rapid orientation to new wards, describe the methods used, and identify facilitating and hindering factors.

**Design:**

Qualitative meta‐synthesis.

**Methods:**

A meta‐aggregation of qualitative literature was conducted, including studies on temporary nursing staff in hospitals.

**Data Sources:**

A database search (MEDLINE, CINAHL) was conducted in July 2021, and updated in April 2024.

**Results:**

Eight studies fulfilled the inclusion criteria and were selected. 10 syntheses from 29 categories based on 115 findings could be formed and divided into two overarching themes: ‘orientation in a new or unfamiliar environment’ and ‘working as agency nursing staff’. The results highlight the importance of rapid orientation for nurses unfamiliar with a ward, and the challenges of collaboration between agency and permanent nurses.

Twelve methods of induction were identified, for example showing around the ward, equipment introduction or use of protocols and folders. Different facilitators were described, like the initiative of the agency nursing staff, a positive attitude of management personnel and a designated contact person. All included studies named a lack of formal feedback mechanisms and time constraints of the permanent staff as barriers to rapid orientation.

**Conclusion:**

This meta‐synthesis emphasises the necessity of induction and rapid orientation for agency nursing staff, demonstrating varied practices. Future concepts should consider both permanent and agency staff perspectives and involve all stakeholders in development, implementation and evaluation.

**Impact:**

A lack of induction of temporary nurses to new wards is discussed as potentially compromising quality of care. The often time‐consuming induction of temporary nurses also poses a challenge for permanent staff. The results of this meta‐synthesis provide an in‐depth insight into the importance of induction and rapid orientation for temporary nursing staff, as well as the factors that facilitate and hinder this.

**Reporting Method:**

The eMERGe reporting guidance.

**Patient or Public Contribution:**

No patient or public contribution.

## 1 Introduction

Internationally, temporary staffing in nursing has ceased to be used to cover temporary peaks in demand, but to compensate for a general shortage of nursing staff (World Health Organization 2020). Following the global nursing shortage, casualization in the nursing workforce has been steadily increasing. This trend has been going on for up to forty years (Boyer 1979; Richardson and Allen 2001; Sheridan, Bronstein, and Walker 1982). The frequency and extent of temporary staffing in nursing are not systematically documented. In the United States (US), about 6% of registered nurses reported currently being a travel nurse (Smiley et al. 2023). A study in the United Kingdom (UK) has found that in all of the studied hospital trusts there was a high level of temporary staffing (over 20%) (Griffiths et al. 2020). In Germany, around 2% of all nursing staff are employed by temporary staffing agencies (Bundesarbeitgeberverband der Personaldienstleister e.V 2023).

However, as a sector in which interpersonal relationships play an important role, ever‐changing nursing staff can be challenging. The evidence of the impact on patient safety outcomes such as falls, infections, medication errors, mortality and length of stay is mixed, with some studies reporting positive effects and others reporting negative or no effects. A systematic review of studies conducted in the US found that negative associations may reflect staffing levels or work environment rather than the specific care by the non‐permanent nurses themselves, whereby the underlying evidence differs widely in terms of the definition of non‐permanent nurses and the study designs (Weerdt et al. 2023). An observational study from the UK, using routinely collected data, indicated that there was no evidence of harm associated with a modest use of temporary nurses so that required staffing levels can be maintained (Dall'Ora et al. 2020).

One factor that is discussed to have an impact on the quality of care provided by staff from outside the ward is induction and rapid orientation on new wards (Dall'Ora et al. 2020; Bae et al. 2010). However, the often time‐consuming familiarisation of temporary nurses with internal procedures also poses a challenge for permanent staff (Aiken et al. 2007). In this US survey, which focused on nurses and their working conditions, permanent nurses reported higher levels of burnout and job dissatisfaction on wards that employed higher numbers of non‐permanent nursing staff (Aiken et al. 2007).

A temporary nurse may feel like a novice nurse, overwhelmed and unprepared for the tasks at hand (Ronnie 2020; Odland et al. 2014), so information about the experiences related to the induction of external staff and which factors might influence its success would help to develop effective induction strategies. Qualitative studies are of particular interest in order to gain a comprehensive insight into the perceived induction and rapid orientation to new wards in the hospital setting. Although qualitative studies on temporary nurses' lived experiences are available (Ronnie 2020; Manias et al. 2003), there is no systematic synthesis on this topic.

## 2 The Review

### 2.1 Objectives

Our main aim was to aggregate the experiences of temporary nursing staff in the hospital setting related to their induction and rapid orientation to new wards, furthermore to describe the methods used, and identify facilitating and hindering factors. For this purpose, the focus was on synthesising existing qualitative studies on the experiences of temporary nursing staff.

The addressed research questions were as follows:

- What are the experiences of temporary nursing staff in the hospital setting related to their induction and rapid orientation?
- What are the methods used, as well as its facilitators and barriers, focusing on the experiences of (i) temporary nurses and (ii) those who work with them?

Because rapid orientation concerns all nursing staff that is new to a ward without being given extra time for induction before having to work as a member of the team, in this paper the term ‘temporary nurses’ includes agency nursing staff as well as pool or float nurses, which are employed by a hospital but work on differing units and thus are permanent to the hospital, but temporary to each ward.

## 3 Methods

### 3.1 Design

The synthesis of qualitative studies followed the approach of meta‐aggregation as it accounts for the nature and traditions of qualitative research whilst reflecting the process of systematic review: it aggregates findings into an overall result that is more than the sum of the individual findings, analogous to meta‐analysis (Lockwood et al. 2024). As an aggregative meta‐synthesis method, meta‐aggregation entails combining the findings of various qualitative studies into themes to produce a general description of the phenomenon (Lockwood et al. 2024; Hannes and Lockwood 2012). For this purpose, based on a systematic literature search, the results of the analysis and interpretation of the data by authors of the included primary studies are synthesised into superordinate statements (Lockwood et al. 2024; Hannes and Lockwood 2012). The approach of meta‐aggregation was chosen, as it enables a synthesis and an understanding of lived experiences without aiming to re‐interpret data but rather to extract generalizable statements in order to deduct practical implications (Lockwood et al. 2024). In this regard, meta‐aggregation differs from other approaches to qualitative evidence synthesis, which focus on re‐interpretation and theory generation rather than aggregation (Lockwood et al. 2024).

The meta‐aggregation was carried out in accordance with the methodological recommendations of the Joanna Briggs Institute (JBI) (Lockwood et al. 2024). The eMERGe reporting guidance (France et al. 2019) was consulted to guide reporting.

### 3.2 Inclusion Criteria

Studies were included in which the focus was on temporary nursing staff in the hospital setting. To cover a diversity of perspectives, articles involved registered nurses, but also nursing assistants or nurses' aides were accepted. The thematic reference to induction and rapid orientation was an inclusion criterion, although this did not have to be the main focus of the study. Research‐based literature with a qualitative design was included, not excluding mixed‐method studies where the qualitative component would have been extracted and handled in the same manner as the purely qualitative research. Literature that adequately reported the sampling strategy and methods of data collection and analysis was considered research‐based (Harris 2011).

We restricted publication language to English or German. Constraints concerning the publication period were not applied.

The inclusion criteria have been framed using the SPIDER tool for qualitative research questions:

- S—Sample: temporary nursing staff (registered nurses, nursing assistants or nurses' aides) or those who work with temporary nursing staff, in the hospital setting.
- PI—Phenomenon of interest: induction, rapid orientation.
- D—Design: interviews.
- E—Evaluation: experiences, methods used, facilitators and barriers (related to induction and rapid orientation).
- R—Research type: qualitative or mixed‐method studies.

### 3.3 Search Methods

MEDLINE (via PubMed) and CINAHL (via EBSCOhost) databases were selected for the systematic literature search. The literature search was conducted in three phases (Lockwood et al. 2024).

#### 3.3.1 Phase 1: Identification of Search Terms

In the first phase, relevant text words were identified via a non‐systematic search of MEDLINE using already known terms. In addition, relevant standardised subject terms for both applied databases were identified via the respective platform‐specific search tools. The PubMed MeSH terms associated with temporary nursing staff were neither specific to nursing nor to the concept of temporary staffing (example: contract services, personnel staffing), so their use was omitted. The corresponding CINAHL headings, however, were related to temporary staffing and thus were applied.

#### 3.3.2 Phase 2: Systematic Database Search

The second phase of the search strategy entails the systematic database search in both databases used with the identified text words and standardised subject terms. The literature thus generated was searched again for further text words and subject headings to create the final search strategy. This entailed search terms related to the main concepts: temporary staff, nurses, the hospital setting and a qualitative design. Search terms of the same concept were combined using the OR Boolean operator, while the results for each concept were then combined using the AND Boolean operator. Language filters for German and English were applied. The final search strategies from both databases are presented in File S1. The database search was completed in July 2021, and updated in April 2024.

#### 3.3.3 Phase 3: Citation Searching

In the third phase of the literature search (citation searching), the reference lists of studies deemed relevant were checked for additional articles. Reference lists of studies that could not be included in the synthesis due to incorrect design or population, but were found to be thematically appropriate, were also checked in this step. In addition, a forward citation search of the included studies was performed using Google Scholar. The citation search was completed in August 2021, and updated in April 2024.

All steps of the systematic literature search were carried out by the first author in close consultation with an experienced expert in health science databases. The data was managed with the research collaboration platform Rayyan (Ouzzani et al. 2016).

### 3.4 Search Outcome

Results from the search were assessed for potential eligibility as defined by the inclusion criteria by title and abstract screening. Potentially relevant studies that met the predefined inclusion criteria after this initial assessment were reviewed in full text. The study selection was conducted by the first author. If there was any ambiguity, a second reviewer was consulted.

### 3.5 Quality Appraisal

A checklist developed specifically for this purpose was used to assess the methodological quality of the included studies (see File S1). The checklist items have been adapted from the Critical Appraisal Skills Programme (CASP) qualitative studies checklist (www.casp‐uk.net), the Oxford Centre for Evidence‐Based Medicine (CEBM) critical appraisal of qualitative studies sheet (www.cebm.ox.ac.uk), and the Consolidated Criteria for Reporting Qualitative Research (COREQ) checklist (Tong, Sainsbury, and Craig 2007). Exclusion of a study due to methodological limitations was not envisaged, as individual findings extracted from the studies were assessed separately for their credibility (Sandelowski and Barroso 2003). In this case, the critical appraisal serves as an overview of the quality of literature included in the review, to support a better overall classification of the results.

Critical appraisal was undertaken by the first author in close consultation with the second author. A consensus was reached on all aspects.

### 3.6 Data Abstraction

To extract findings from the results of the included studies a standardised tool was used, whereby a finding in the context of meta‐aggregation is defined as a verbatim extract of the primary study author's analytic interpretation of their results or data (Lockwood et al. 2024). Since it was expected that our research question would not also be the research question of the included studies, but that there would merely be overlap, the categories and themes of the original papers were not scored as findings themselves, but were searched for statements and findings, which were then extracted as findings. Each extracted finding is to be accompanied by an illustration from the same text that substantiates the finding, either as a direct quotation of the participants' voices, fieldwork observations or other supporting data (Lockwood et al. 2024). This process was repeated by reading the full texts until no further findings could be extracted.

For each extracted finding, a level of credibility was allocated. This was completed in the data extraction table with each finding and its accompanying illustration, if any (Lockwood et al. 2024). The three levels of credibility are:

- ‘unequivocal’ findings, which leave no reasonable doubt and are supported by clear, verbatim quotes from interviewees.
- ‘credible’ findings, which may not be clearly attributable to quotes or sources.
- ‘unsupported’ findings, which cannot be found in the data presented by the publication.

In the meta‐aggregation, all unequivocal and credible findings were included, while credible findings were marked as such. Unsupported findings were listed separately (Lockwood et al. 2024). This process was undertaken iteratively by the first author and discussed with and reviewed by the second author.

### 3.7 Synthesis

After the extraction of all findings from all included studies, data synthesis in meta‐aggregative reviews requires two steps (Lockwood et al. 2024):

- The development of ‘categories’, where a category is a brief description of a key concept arising from the aggregation of two or more like findings.
- The development of one or more ‘synthesised findings’, where a synthesised finding is an overarching description of a group of categorised findings.

Our categories were formed by combining extracted findings that were similar in their concepts, rather than in their wording. This was done regardless of the categories formed in the included primary studies. To avoid losing uniqueness and meaningfulness, the categories and syntheses were formulated in complete sentences rather than keywords (Hannes and Lockwood 2012).

Additionally, data concerning the methods of rapid orientation and facilitators and barriers to induction of temporary nursing staff were extracted and tabulated.

Data analysis and aggregation were carried out by the first author, supervised by the second author. Both authors discussed all resulting syntheses; if no consensus was reached, a third person was involved.

### 3.8 Trustworthiness

Trustworthiness of this meta‐synthesis is based on the four criteria described by Lincoln and Guba (Lincoln and Guba 1985): credibility, confirmability, dependability and transferability.

The first author worked as a nurse in temporary employment, and therefore understands the importance of rapid induction for temporary nurses from their perspective. This orientation to the situation and the context facilitates credibility. At the time of conduction of this meta‐synthesis, the author had stopped working for a temporary nursing agency. However, to enhance confirmability as well as dependability, all findings were finally evaluated by a third person (registered nurse, master's student in nursing and health sciences), who was not involved in the research process. To address transferability, we provide the data aggregation process as a supplementary file, which allows readers to assess the applicability of the presented findings in other contexts.

## 4 Results

### 4.1 Study Inclusion

Through the database search (*n* = 1757) and additionally identified literature (*n* = 67), a total of 1566 articles were screened after exclusion of duplicates. After reading 53 full texts, eight studies could be included. The update of the literature search resulted in 434 new records, three of which were assessed for eligibility in full text, but no additional study could be included.

Forty eight studies that were read in full were excluded for not having a qualitative design (*n* = 27), not focusing on temporary nursing staff or those who work with them (*n* = 10) or having a focus other than the experiences of their subjects (*n* = 11).

**FIGURE 1:**
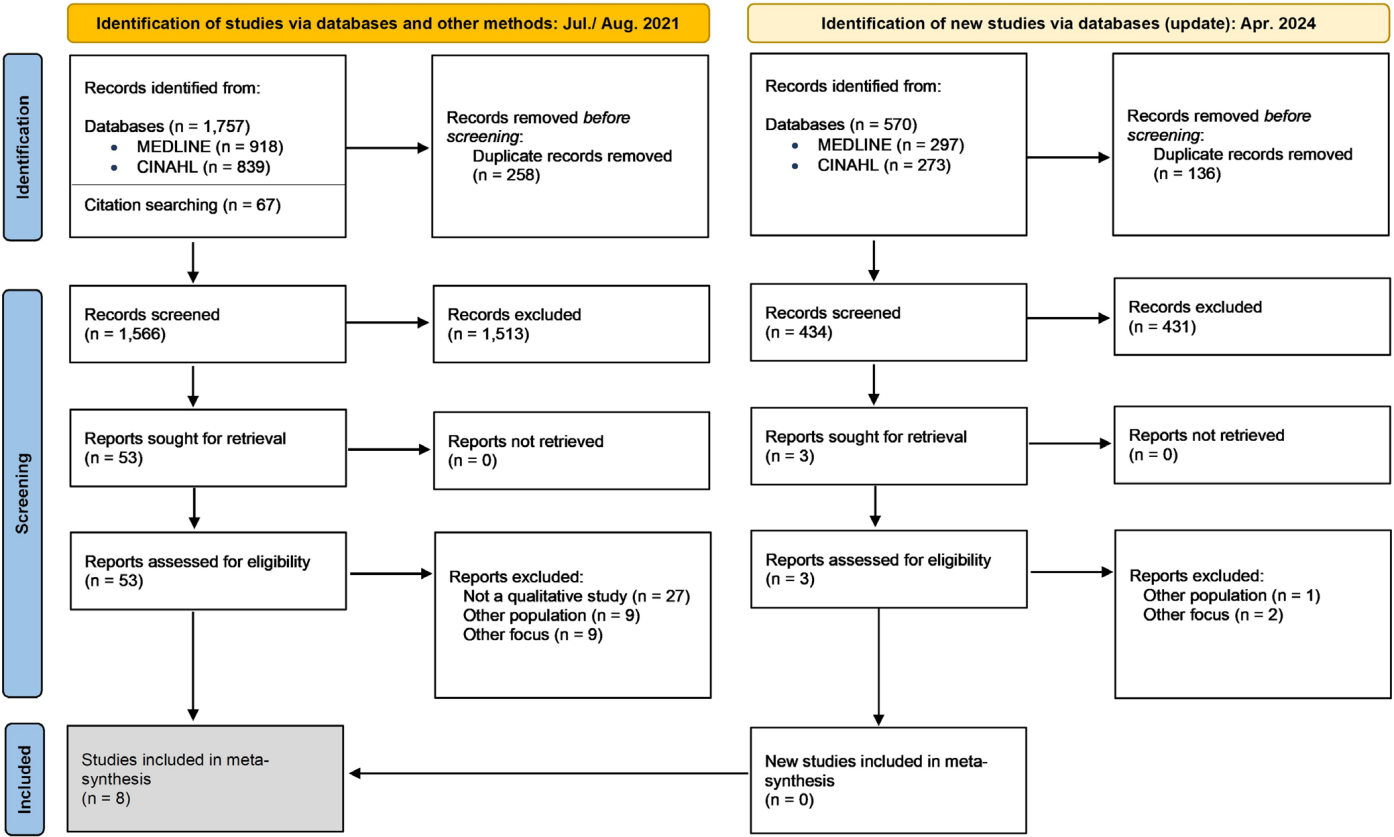
Flowchart of article selection process.

The article selection process is summarised in Figure 1. For excluded full texts see File S1.

### 4.2 Characteristics of Included Studies

The included studies date from the years 2003 to 2020 and were conducted in South Africa (Ronnie 2020; Collier 2011; Muller 2014), Australia (Manias et al. 2003; FitzGerald et al. 2007), Germany (Krebs, Hasseler, and Lietz 2020), Sweden (Berg Jansson and Engström 2017) and the United Kingdom (Hass, Coyer, and Theobald 2006). Table 1 provides a summary of the eight articles included, giving information on study participants and methods. All studies interviewed agency nursing staff, with the exception of one study that interviewed members of staff representation, nursing management and human resources administration (Krebs, Hasseler, and Lietz 2020). On par with the predefined criteria, this study was included because it focused on those who worked with temporary nursing staff, and their perspectives on temporary nursing. In one study, some participants also worked as midwives (Manias et al. 2003), and another study additionally interviewed nurses in permanent ward positions (Berg Jansson and Engström 2017). Three studies also interviewed agency nursing aides or assistants (Ronnie 2020; Muller 2014; FitzGerald et al. 2007). Since the included studies exclusively address registered agency nurses and agency nursing assistants, the term agency nursing staff is used in the following instead of temporary nursing staff.

**TABLE 1:** Summary of included articles.

Author(s), year	Country	Methods	Setting	Participants
Berg Jansson and Engström 2017	Sweden	Individual interviews	Critical care unit	5 registered agency critical care nurses, 5 registered critical care nurses
Collier 2011	South Africa	Individual interviews	Critical care unit	10 registered agency critical care nurses
FitzGerald, et al. 2007	Australia	Focus group interviews	Intensive care unit	40 registered agency nurses, 21 agency nursing assistants
Hass et al. 2006	United Kingdom	Individual interviews	Intensive care unit	8 registered agency intensive care unit nurses
Krebs, Hasseler and Lietz 2020	Germany	Individual and focus group interviews	Unnamed	11 ward nurse managers, coordinators of the nursing pool, hospital nurse directors, members of the employee representation and members of human resources
Manias et al. 2003	Australia	Individual interviews	General ward	10 registered agency nurses (some participants also worked as midwives)
Muller 2014	South Africa	Individual interviews	General ward and intensive care unit	5 registered agency nurses, 8 agency nursing assistants
Ronnie 2020	South Africa	Individual interviews	Intensive care unit	2 registered agency nurses, 9 agency nursing assistants

### 4.3 Quality Appraisal of the Included Studies

Eight items were used to assess the methodological quality of the included studies. Overall, methodological quality of the included studies was found to be good, detailed in File S1. Strengths across the studies included presenting a clear statement of the research aims and having appropriate data collection methods. The most frequent limitations were lacking clarity regarding a sufficiently rigorous data analysis (Ronnie 2020; Manias et al. 2003; Collier 2011; Muller 2014; FitzGerald et al. 2007; Berg Jansson and Engström 2017), and an insufficient description of the study sample (FitzGerald et al. 2007; Krebs, Hasseler, and Lietz 2020; Hass, Coyer, and Theobald 2006).

### 4.4 Findings of the Review

A total of 109 ‘unequivocal’ (*n* = 50) or ‘credible’ (*n* = 59) findings could be extracted (see File S2). From these, 29 categories were formed via similarities in content out of which ten syntheses were generated. Four categories resulted exclusively from ‘credible’ findings. Six findings were classified as ‘unsupported’, but these could not be synthesised. The ten syntheses were divided into two overarching themes: ‘orientation in a new or unfamiliar environment’ and ‘working as agency nursing staff’.

The overarching themes and syntheses are summarised below; all underlying categories together with the number of supporting findings and references of the corresponding studies are displayed in Table 2. A detailed listing of the syntheses, their respective categories and their respective findings (including their bibliographic references, supporting quotations and levels of credibility) is presented in File S3 ‘Process of data aggregation’.

**TABLE 2:** Overview of categories, syntheses and overarching themes.

Number of findings [study references]	Categories	Syntheses
Overarching theme 1: orientation in a new or unfamiliar environment		
6 (Manias et al. 2003; Collier 2011; FitzGerald et al. 2007; Krebs, Hasseler, and Lietz 2020; Hass, Coyer, and Theobald 2006)	Orientation is pertinent to agency nursing staff	Agency nursing staff requires orientation on each new ward in order to do their job
2 (Hass, Coyer, and Theobald 2006)	Knowing the equipment plays a role in keeping patients safe	
3 (FitzGerald et al. 2007; Krebs, Hasseler, and Lietz 2020)	Each ward is different	
3 (FitzGerald et al. 2007; Hass, Coyer, and Theobald 2006)	Familiarity with the ward can boost efficiency of agency nursing staff	
5 (Collier 2011; Krebs, Hasseler, and Lietz 2020; Berg Jansson and Engström 2017)	Permanent staff plays an important role in providing orientation for agency nursing staff	Agency nursing staff relies on permanent staff to be prepared for orientation and induction
2 (Berg Jansson and Engström 2017)	Clear documentation is a prerequisite for agency nursing work^a^	
9 (Manias et al. 2003; Krebs, Hasseler, and Lietz 2020; Berg Jansson and Engström 2017; Hass, Coyer, and Theobald 2006)	Written orientation and protocols work as a facilitator of agency nursing work	
6 (Ronnie 2020; Manias et al. 2003; Krebs, Hasseler, and Lietz 2020)	Often, there is not sufficient time for orientation	Time constraints and lack of routines may hinder effective orientation
6 (Manias et al. 2003; Hass, Coyer, and Theobald 2006)	Lack of uniform orientation routines may hinder effective induction	
2 (Krebs, Hasseler, and Lietz 2020; Hass, Coyer, and Theobald 2006)	Making key staff members known to agency nursing staff may be helpful in orientation^a^	Individualised orientation aided by key staff members may be helpful to agency nursing staff
4 (Muller 2014; Krebs, Hasseler, and Lietz 2020)	Orientation should be flexible to adapt to individual nurse's needs	
Overarching theme 2: working as agency nursing staff		
2 (Collier 2011; FitzGerald et al. 2007)	Agency nursing staff may not know what they missed in terms of patient care^a^	Agency nursing staff may require patience, time and extra information to care for patients safely
4 (Manias et al. 2003; FitzGerald et al. 2007)	Detailed patient reports are necessary for agency nursing staff to deliver adequate care	
3 (Ronnie 2020; Muller 2014)	Agency nursing staff may require extra time to learn new skills	
2 (FitzGerald et al. 2007; Krebs, Hasseler, and Lietz 2020)	Agency nursing staff being given a chance to continue their care decreases their need for orientation	
2 (Ronnie 2020; FitzGerald et al. 2007)	Agency nursing staff may feel that patient allocation by permanent staff is unjust	Interactions between agency and permanent nurses may be difficult or conflict ridden, depending on support of nursing management
6 (Ronnie 2020; Krebs, Hasseler, and Lietz 2020)	Agency nursing staff is confronted with differing and partially unrealistic expectations	
6 (Ronnie 2020; Muller 2014; Berg Jansson and Engström 2017)	Shift and ward managers are responsible for creating a constructive workplace culture	
5 (Manias et al. 2003; Collier 2011; Berg Jansson and Engström 2017)	In a new environment, agency nursing staff may feel vulnerable or alone	The level of support offered by permanent staff can influence how respected or insecure agency nursing staff feels
3 (Manias et al. 2003; Collier 2011)	Agency nursing staff experiences varying levels of support from permanent staff	
3 (Collier 2011; FitzGerald et al. 2007; Krebs, Hasseler, and Lietz 2020)	Agency nursing staff felt supported when permanent staff showed respect and shared their knowledge	
4 (Krebs, Hasseler, and Lietz 2020)	Agency nursing staff does not always bring the relief to permanent staff that they are intended for	Ever‐changing agency nursing staff may pose as a burden on permanent staff
2 (Krebs, Hasseler, and Lietz 2020; Berg Jansson and Engström 2017)	Working with constantly new agency nursing staff can be stressful to permanent staff	
2 (Ronnie 2020; Hass, Coyer, and Theobald 2006)	Agency nursing staff values feedback	The lack of feedback mechanisms for and from agency nursing staff may hinder professional development and collaboration
5 (Manias et al. 2003; Collier 2011; FitzGerald et al. 2007; Hass, Coyer, and Theobald 2006)	A lack of formal feedback mechanisms may hinder the giving and receiving of honest and constructive feedback	
3 (Manias et al. 2003; Krebs, Hasseler, and Lietz 2020)	Feedback concerning individual agency nurses can be used to exclude them from ward rotation^a^	
3 (FitzGerald et al. 2007; Krebs, Hasseler, and Lietz 2020; Hass, Coyer, and Theobald 2006)	Some agency nursing staff has developed an own routine to orient themselves	Agency nursing staff needs to be proactive, confident and self‐reliant
3 (Manias et al. 2003; Collier 2011; FitzGerald et al. 2007)	Agency nursing staff is responsible for filling their own deficits in knowledge	
3 (Ronnie 2020; Krebs, Hasseler, and Lietz 2020; Hass, Coyer, and Theobald 2006)	Confidence and openness are prerequisites for working as an agency nurse	

^a^ Category results exclusively from ‘credible’ findings.

### 4.5 Overarching Theme 1: Orientation in a New or Unfamiliar Environment

Orientation to any new ward by regular nursing staff or shift and ward managers using documentation and written orientation concepts and protocols was considered essential by the interviewees. However, hindering factors were lack of time resources of the permanent staff and lack of orientation concepts and routines.

#### 4.5.1 Synthesis 1: Agency Nursing Staff Requires Orientation on Each New Ward in Order to Do Their Job

The relevance of rapid orientation for agency nursing staff is mentioned in each of the included studies, as being pertinent to their work on each new ward. Because each new ward is different, rapid briefing and orientation of non‐departmental nursing staff is considered a basic requirement for agency nursing staff to work safely and efficiently. This includes familiarisation with the equipment, processes and the ward itself. This was not only said to positively influence patient safety, but also boost agency nurses' efficiency.

‘I need to know where certain things are so that my patient is safe and that is, you know, the arrest trolley and where I can do gases and where the suction equipment is, oxygen […] and then I'm happy’. Verbatim participant quote (Hass, Coyer, and Theobald 2006)

This synthesis is based on 14 findings grouped into four categories (see Table 2 and File S3).

#### 4.5.2 Synthesis 2: Agency Nursing Staff Relies on Permanent Staff to Be Prepared for Orientation and Induction

Nursing staff from outside the ward are dependent on the permanent staff. Through good documentation, personal helpfulness and written orientation concepts, permanent staff are expected to be prepared for working with external staff. Written concepts may include orientation packages explaining the layout of the ward, procedures and processes or information on medication. It was said to be the staff nurse's responsibility to not only prepare these written instructions, but also to be open and considerate to questions and need for support.

‘They [permanent staff] are quite accommodating when you come in as an agency nurse and they help you to adapt to your shift because they would like to have you there, that's wonderful’. Verbatim participant quote (Collier 2011)

This synthesis is based on 16 findings grouped into three categories (see Table 2 and File S3).

#### 4.5.3 Synthesis 3: Time Constraints and Lack of Routines May Hinder Effective Orientation

Lack of time and lack of orientation concepts are named as barriers that can make orientation and rapid induction more difficult. Permanent nursing staff is generally expected to take over the orientation of agency colleagues in addition to their nursing work, which is often hindered by a constantly high workload. Orientation was said to be very different on each ward, with wards that frequently use external staff having been said to offer more effective induction concepts, while hospitals inexperienced with external staff and a lack of routine in working with them, would offer less effective orientation.

‘Sometimes the [permanent] nurses haven't got the time or the resources to have someone around to ask questions. They are just too busy’. Verbatim participant quote (Manias et al. 2003)

This synthesis is based on 12 findings grouped into two categories (see Table 2 and File S3).

#### 4.5.4 Synthesis 4: Individualised Orientation Aided by Key Staff Members May Be Helpful to Agency Nursing Staff

This synthesis describes the benefits of individualised rapid orientations, highlighting the importance of key staff members in leadership roles such as shift and ward managers. Pre‐appointed contact persons who are prepared for the questions and needs of nurses from outside the ward and who are assigned to them at the beginning of the shift are considered very helpful. Ideally the prior knowledge of agency nursing staff should be taken into account for an individualised orientation. The extent to which agency nurses received orientation to new wards varied widely. While some orientation programmes were offered at hospital level and spanned over two to three days, other interviewees described being left to orient themselves on a new ward. Overall it was said that ward nurses greatly shaped the experience of their agency colleagues.

‘It was very good I found some support from the staff they were good were keen to show me everything […]’. Verbatim participant quote (Muller 2014)

This synthesis is based on six findings grouped into two categories (see Table 2 and File S3).

### 4.6 Overarching Theme 2: Working as Agency Nursing Staff

The focus in this overarching theme is the cooperation within the nursing team as well as the prerequisites for working as agency nursing staff. Positive attitudes and respectful behaviour on the part of management personnel towards nursing staff from outside the ward were seen as conducive, while a lack of feedback mechanisms and unrealistic expectations were considered to make collaboration more difficult.

#### 4.6.1 Synthesis 5: Agency Nursing Staff May Require Patience, Time and Extra Information to Care for Patients Safely

Both detailed handover reports and additional time are listed as prerequisites for agency nursing staff, which must be fulfilled for high‐quality patient care and without which missed nursing care cannot be ruled out. Here interviewees pointed out that they may not know what they might have missed in terms of patient care, as they may have been wholly unaware of certain tasks or responsibilities. On the other hand, it was considered helpful by agency nursing staff to be able to care for the same group of patients several shifts in a row, also to help decrease their need for extra orientation. Even experienced nurses are confronted with new tasks when working on unknown wards, such as the operation of dialysis machines. If agency nursing staff is not provided with additional time and instructions to learn new skills, individual task complexes may not be completed and a sense of incompetence and dissatisfaction may arise.

‘When I ask for information about a patient's past history so I can care for them better, I usually don't get the right information. I usually just get the comment, “You just have to do this thing,” rather than answer my question so I can make my own decision on what I am going to do. I feel that they don't really let me know the patient's holistic picture because they want me to do a series of tasks’. Verbatim participant quote (Manias et al. 2003)

This synthesis is based on 11 findings grouped into four categories (see Table 2 and File S3).

#### 4.6.2 Synthesis 6: Interactions Between Agency and Permanent Nurses Maybe Difficult or Conflict Ridden, Depending on Support of Nursing Management

This synthesis represents the influence of nursing ward and shift managers on the conflict‐prone working climate between regular and agency nursing staff. Nurse managers are said to play a key role in shaping the ward culture and attitude towards agency nurses. They could counteract the feeling of agency nursing staff of being treated unfairly or being subjected to unrealistic expectations by creating a respectful and helpful working climate through their own example. The expectations placed on the agency nursing staff present themselves as very different and partially unrealistic. For example, experienced nurses noted that they felt insecure on new wards and in new specialties and would need professional instruction. Also, with regard to basic nursing activities, it cannot be expected to know the premises and special features of individual wards without prior briefing. Creating appropriate expectations and a positive working atmosphere is seen as the responsibility of the nursing managers. By being respectful, responsive and open to questions, and providing a motivational leadership style by setting a positive example, nurses felt more secure and supported. Otherwise, frustration about poor team interaction and lack of communication set in. This also pertained to patient allocation. Some agency nurses mentioned that patient allocation may have been unjust, having been allocated especially care intense patients instead of the same patients for each consecutive shift. This, too, was seen as the responsibility of ward or shift managers.

‘[…] for the unit to work to function well it is the leader first to respect the agency staff I have seen this if she is not weighing them at the same level the permanent staff think they are supers of the agency staff irrespective of how senior you are to them […]’. Verbatim participant quote (Muller 2014)

This synthesis is based on 14 findings grouped into three categories (see Table 2 and File S3).

#### 4.6.3 Synthesis 7: The Level of Support Offered by Permanent Staff Can Influence How Respected or Insecure Agency Nursing Staff Feels

The picture painted by nursing staff from outside the ward with regard to cooperation with the permanent staff varied greatly. The feeling of being left alone, being vulnerable or not respected arose when permanent staff did not show themselves to be approachable or helpful. A lack of responsiveness and willingness to help on the part of the permanent nursing staff could be due to the high workload and lack of time, according to the agency nursing staff. Agency nurses described vastly varying levels of support, from ward nurses being described as stand‐offish to being friendly and supportive. If permanent staff shared their knowledge and offered support, their colleagues from outside the ward felt respected and welcome.

‘If you look at a new agency nurse, somebody that comes into a new situation, how vulnerable they feel and the unit is not known, they don't know the staff. Now they don't actually know where they stand and then you find that person is vulnerable because where is the support you want’. Verbatim participant quote (Collier 2011)

This synthesis is based on 11 findings grouped into three categories (see Table 2 and File S3).

#### 4.6.4 Synthesis 8: Ever‐Changing Agency Nursing Staff May Pose as a Burden on Permanent Staff

Agency nursing staff, who are unfamiliar with the ward and internal procedures, have lower work efficiency and require constant instruction and supervision; a task that permanent staff need to carry out in addition to their regular workload. The continuous training of new colleagues, coupled with the many questions they may have, can be perceived as a burden by permanent nursing staff. This may mean, that agency nurses are not always perceived as a relief, but might be seen as an additional stress factor.

‘Since we have patients that are really critically ill, then it's reassuring to know that I know what colleagues are with me, and who can do what. It's not that I don't want to be with new [critical care nurses], but you know what it's like, I think it's a bit stressful [to deal with temporary critical care nurses]’. Verbatim participant quote of a permanent critical care nurse (Berg Jansson and Engström 2017)

This synthesis is based on six findings grouped into two categories (see Table 2 and File S3).

#### 4.6.5 Synthesis 9: The Lack of Feedback Mechanisms for and From Agency Nursing Staff May Hinder Professional Development and Collaboration

In this synthesis, the importance of constructive feedback both from and for agency nursing staff becomes clear. However, as described in all included studies, a lack of formal feedback mechanisms hinders continuous development of oneself, one's nursing practice, or a unit. Agency nurses described valuing all feedback, positive or negative, as a means for professional improvement, pointing out that their only source of feedback on their nursing work could come from ward nurses and managers. Yet some agency nursing staff mentioned the sole form of personal feedback as having been blocked from specific assignment sites, while also pointing out that barriers to give feedback could be high as well, with agency nurses fearing personal or professional repercussions if giving negative feedback directly to nurse managers. This may be a loss for wards and ward staff, as agency nurses could bring new insights and constructive criticism.

‘It is great to get feedback as that is the only way you can improve. If I am better at what I am doing then my patient is going to benefit at the end of the day. It doesn't matter if it is positive or negative, it is still feedback’. Verbatim participant quote (Hass, Coyer, and Theobald 2006)

This synthesis is based on 10 findings grouped into three categories (see Table 2 and File S3).

#### 4.6.6 Synthesis 10: Agency Nursing Staff Needs to Be Proactive, Confident and Self‐Reliant

It becomes clear that agency nurses are required to be confident, proactive and acquire their own routines in order to be able to work independently on new wards as quickly as possible. This also refers to knowing and asserting one's own professional boundaries. The routines that help them orientate themselves quickly on new wards include observing their colleagues, independently reading up on information or arriving early at the ward to familiarise themselves with the premises before starting their shift. It was also described that this kind of proactive behaviour was carried out so as not to bother or be perceived as a burden by permanent nursing staff and aid integration into the nursing team.

‘I think it's your own responsibility to make sure you are on top of things, and if you are not then to find out or access people who can get you up‐to‐date. Only you would know what your deficits are’. Verbatim participant quote (Manias et al. 2003)

This synthesis is based on nine findings grouped into three categories (see Table 2 and File S3).

### 4.7 Unsupported Findings

The six findings that were classified as ‘unsupported’ in the credibility assessment (see File S2) fall into two categories.

- Effective orientation for agency nursing staff should be well conceptualised by the ward or hospitals: The importance of effective orientation for agency nursing staff is again underlined, with the suggestion to organise this centrally with the use of e‐learning, and considering previous experience of the nurses. This category was comprised of two findings.
- Strong networks and communication between all stakeholders of agency nursing are necessary for teams to function well: The four findings in this category emphasise on the one hand the importance of formal feedback mechanisms and good cooperation between the stakeholders of agency nursing, but at the same time note that these factors could be improved. A suggestion for improvement would be to limit the number of sites to which agency nursing staff are dispatched.

### 4.8 Synopsis of Methods of Rapid Orientation and Induction

‘Good hard information at your fingertips is what you need when you can't come away from the bedside, and when you are new or when you are an agency nurse’. Verbatim participant quote (Hass et al. 2006)

Twelve different methods of induction and rapid orientation of agency nursing staff were mentioned in the included studies, with no visible pattern or clustering of a single method. A summary of the different methods of induction and orientation can be found in Table 3. Documentation, such as handover sheets and patient reports or charting, is mentioned as a prerequisite for the work of agency nursing staff. Written briefings in the form of protocols, written procedures and ward folders were also named as an important method of briefing, as mentioned in four studies (Manias et al. 2003; Krebs et al. 2020; Berg Jansson and Engström 2017; Hass et al. 2006). Some form of unstructured brief orientation by regular staff on procedures or the ward setup itself was cited in six of the eight studies (Ronnie 2020; Manias et al. 2003; Collier 2011; Muller 2014; FitzGerald et al. 2007; Hass et al. 2006). Of these, three studies also cite an unstructured orientation that refers to a rapid technical or personnel briefing rather than a showing of the premises (Ronnie 2020; Collier 2011; Muller 2014). Structured briefings on equipment, documentation or contingency plans were mentioned in three studies (Manias et al. 2003; Muller 2014; Krebs et al. 2020).

**TABLE 3:** Overview of methods of induction and rapid orientation.

Methods of induction and rapid orientation	Reference							
	Berg Jansson and Engström 2017	Collier 2011	FitzGerald et al. 2007	Hass et al. 2006	Krebs et al. 2020	Manias et al. 2003	Muller 2014	Ronnie 2020
Orientation through permanent staff								
Unstructured orientation of the premises on site		X					X	X
Unstructured orientation of the rooms on site		X	X	X		X	X	X
Separate, verbal handover			X					X
Structured orientation								
Device briefings					X			
Documentation briefing					X	X	X^a^	
Briefing on emergency protocols						X		
Self‐initiative of non‐permanent nurses								
Independent orientation at ward level				X	X			
Ad‐hoc questions			X					X
Written induction								
Written protocols	X			X^b^	X			
Written ward procedures	X			X	X^c^	X		
Other								
Short written briefing by nursing agency						X^d^		
Attendance at briefing programs for newly hired permanent staff							X	

^a^ Two days, unpaid.

^b^ Example: nutrition, pharmaceuticals.

^c^ Brief instructions and flowcharts.

^d^ Information on code of conduct, uniforms, company regulations, sick leave, hospital location, parking, systems of care, procedures for emergencies.

### 4.9 Synopsis of Facilitators and Barriers of Rapid Orientation and Induction

‘The permanents shouldn't look down upon us, like we don't know anything. Talk to us as human beings. Talk to us, tell us what to do. We do have feelings’. Verbatim participant quote (Ronnie 2020)

An overview of the enabling and hindering factors of rapid orientation identified in the studies is presented in Table 4. Facilitating factors that were frequently cited include providing designated contact persons for agency nursing staff (Ronnie 2020; Manias et al. 2003; Krebs et al. 2020; Hass et al. 2006) and positive attitudes and respectful treatment of agency staff by nurse leaders (Ronnie 2020; Muller 2014; Berg Jansson and Engström 2017). Self‐confidence and initiative on the part of agency nursing staff were cited in five studies as personal characteristics conducive to induction and orientation (Manias et al. 2003; Collier 2011; FitzGerald et al. 2007; Krebs et al. 2020; Hass et al. 2006). Continuity of care, whether by being able to care for the same group of patients again in the follow‐up shift (FitzGerald et al. 2007) or avoiding a change of assignment sites (Krebs et al. 2020; Hass et al. 2006), was seen as a facilitating factor, while frequent ward changes (Ronnie 2020; Collier 2011) or frequent changes of agency nursing staff (Krebs et al. 2020) were mentioned as hindering factors. In terms of hindering factors, all studies named both lack of feedback mechanisms and lack of time of permanent staff as hindering rapid orientation. Differences in ward setup and equipment at each ward were also cited as hindering factors (Ronnie 2020; Muller 2014; FitzGerald et al. 2007; Krebs et al. 2020; Hass et al. 2006).

**TABLE 4:** Overview of facilitators and barriers to induction of agency nursing staff.

Facilitators (+)/barriers (−)	Berg Jansson and Engström 2017	Collier 2011	FitzGerald et al. 2007	Hass et al. 2006	Krebs et al. 2020	Manias et al. 2003	Muller 2014	Ronnie 2020
Hospital level								
+								
Functioning feedback mechanisms		X						
Involving nurses from outside the ward in decision‐making processes			X					
Individually adaptable induction					X		X	
−								
Lack of feedback mechanisms	X	X	X	X	X	X	X	X
Lack of routine in dealing with staff from outside the ward				X				
Unpaid induction programs							X	
Ward level								
+								
Good documentation	X							
Clear, labelled storage of equipment and supplies	X							
Additional, verbal patient information			X					
−								
The spatial differences of each ward			X		X			
The different equipment of each ward				X			X	X
Insufficient documentation			X			X		
Lack of a uniform induction concept					X			
Overestimation of previous induction						X		
Too high a quota of non‐permanent nurses	X							
Prerequisites of the permanent staff								
+								
Positive attitude of the management	X						X	X
Positive attitudes of permanent staff			X					
Designated contact persons		X		X	X	X		
Preparedness of shift supervisors for working with non‐station nurses					X			
Patience of permanent staff		X					X	
Sharing of knowledge with non‐permanent nursing staff	X						X	
Positive attitude of the management	X						X	X
−								
Lack of time of permanent staff	X	X	X	X	X	X	X	X
Unfair patient allocation		X	X					X
Unrealistic expectations					X			X
Prerequisites of the agency staff								
+								
Personal initiative		X	X	X	X			
Self‐confidence				X	X	X		
Outgoingness		X	X					
−								
Feeling of not belonging to a team		X						
Frequent ward changes		X						X
After the shift								
+								
Being able to care for the same group of patients repeatedly			X					
More frequent assignments to the same ward/hospital				X	X			
Frequent change of external staff	X				X			

## 5 Discussion

Our objective was to conduct a meta‐aggregation on temporary nurses' experiences related to their induction and rapid orientation to new wards in the hospital setting, and the factors that facilitate and hinder this. However, since only studies that considered ‘agency nursing staff’ or those who worked with ‘agency nursing staff’ could be identified, this term was used for the meta‐aggregation.

Ten syntheses were formed from eight included studies on the overarching themes of ‘orientation in a new or unfamiliar environment’ and ‘working as agency nursing staff’. Our results highlight the importance of induction and rapid orientation for nursing staff not familiar with a ward, which often falls short due to lack of routine or time, while simultaneously underpinning the vital role of a wards culture for the experience and self‐perceived efficacy of agency nursing staff. In the following, we discuss our main results against the background of international research, structured according to our research questions.

### 5.1 The Experiences of Agency Nursing Staff

The experiences reported by agency nursing staff on the topic of induction and rapid orientation varied widely in our study, revealing divergent perceptions regarding the support they received from permanent staff. The quality of collaboration between permanent and agency nursing staff was significantly shaped and exemplified by the attitude of nurse leaders towards temporary staff.

The results of other reviews confirm that temporary nurses are dependent on their permanent colleagues, as they have no knowledge of the structures, processes and special features of the new locations and therefore rely on their permanent colleagues for orientation and induction (Birmingham et al. 2019). Miscommunication and unrealistic expectations on both sides can make collaboration difficult (Simpson and Simpson 2019). Temporary nurses stated that they felt left alone and had little support in their induction and in patient care (Birmingham et al. 2019), while the feeling of being isolated or feeling like an outsider was described in our results as well as in another review (Simpson and Simpson 2019).

Consistent with our results, 46 temporary nurses who were interviewed within a phenomenological study (Lapeña‐Moñux et al. 2014) described feelings of anxiety, stress or uncertainty when the change to a new ward was imminent. This was often due to the fact that they were not given time to orient themselves and that their permanent colleagues met them with a lack of understanding. Moreover, lack of induction in the processes and devices resulted in a loss of professional self‐confidence (Lapeña‐Moñux et al. 2014).

### 5.2 Methods, Facilitators and Barriers to the Induction of Agency Nursing Staff

In our meta‐synthesis, in both the extracted methods of induction and their facilitating factors, no visible pattern or clustering could be identified. Induction can be given on site, for example by showing the premises or equipment, or in written form of protocols or manuals. Thematically related studies that overlap in topic but are based on different research perspectives, report on similar results. One integrative literature review (Birmingham et al. 2019) found that non‐permanent nursing staff required a written orientation package, clear guidelines, and support from regular staff and nursing management to do their jobs safely and effectively. Both studies (Manias et al. 2003; Hass et al. 2006) included there were also included in our meta‐synthesis.

Of the 17 facilitators to induction mentioned in the included studies, none were reported in more than five studies. The initiative of agency nurses and positive interaction with them on the part of nurse leaders as well as designated contact persons were mentioned slightly more frequently. Five of our included studies reported personal initiative, self‐confidence and outgoingness as personal prerequisites that agency nursing staff should have. We could not identify any additional literature on these requirements.

A survey (Hoffman and Sadovszky 2018) examined 220 nurses about the types of support they needed when assigned to unfamiliar wards. The most common responses were a designated contact person on the ward and written briefings. However, problems arose frequently when designated contact persons did not have time to answer questions, and the written information had not been tailored to the needs of the temporary nurses. Therefore, the temporary nurses expected additional support from ward managers (Hoffman and Sadovszky 2018). Based on a literature review (Mazurenko et al. 2015), its authors urge hospitals to better prepare their leadership staff for the collaboration with agencies and temporary staff and to provide systematic induction programmes.

The hindering factors provide a clearer picture. In all studies, a lack of feedback mechanisms and a lack of time on the part of the permanent staff were mentioned as having a negative influence on the induction. Our results also show the sometimes conflict‐ridden relationship between temporary and permanent nurses, which has been frequently reported on (Lapeña‐Moñux et al. 2014; Bajorek and Guest 2019; Batch and Windsor 2015). Poor communication between temporary nursing agencies and hospitals, as well as between temporary and permanent staff intensify the tenseness of the relationship, which is exacerbated further by inadequate organisational structures (Batch and Windsor 2015). In an interview study (Bajorek and Guest 2019) it was noted that temporary caregivers can represent an additional burden for their permanent colleagues, as temporary staff unfamiliar with the ward always require supervision. This is in line with our results that interactions between agency and permanent nurses may be difficult, depending on support of nursing management. Different hospitals have published about various methods of induction and rapid orientation (Crowell‐Grimme and Garner 2007; Good and Bishop 2011; Leon and Pase 2004). However, none have focused on the inter‐staff relationship or ward culture aspects of collaboration between permanent and temporary staff.

Our findings are largely in line with the international research results. However, this meta‐synthesis also offers a list of precise measures for induction and rapid orientation based on the literature, with their facilitating and hindering factors. The specification and detailed listing of the influencing factors seems to be a new approach that could not be determined in this form in the existing literature.

### 5.3 Strengths and Limitations

As in all reviews, even the systematic steps in the literature search process cannot guarantee that no relevant articles were missed (Hannes and Lockwood 2012), particularly since the literature search was limited to English and German language studies. Despite the large number of syntheses, it cannot be ruled out that data saturation was not reached.

The necessary comparability of the included studies, as required by the method of meta‐aggregation (Hannes and Lockwood 2012), also must be discussed. The countries of origin and their respective health care systems vary. Nonetheless, all countries are comparable as developed countries (United Nations Conference on Trade Development 2019), with the exception of South Africa, which nevertheless has a stable and sophisticated health care system (Villiers et al. 2020). However, whether our findings can be applied to other health care systems must be assessed in each individual case. Both the original research aims of the included studies and the participants differ, portraying perspectives of different professional roles within patient care (registered nurses, nurses' aides and both nurses and midwives) or adjacent responsibilities (management, members of staff representation, administration). Some included studies did not differentiate between professional roles, thereby making it impossible to make clear distinctions in this meta‐synthesis. Seeing as the findings of these differing studies were still able to form categories and syntheses, and thus support each other's statements, the diversity of perspectives could be viewed as a strength of this meta‐synthesis.

### 5.4 Implications for Practice and Further Research

This meta‐synthesis underlines the indispensability of induction and rapid orientation for agency nursing staff in the hospital setting, and at the same time demonstrates how differently this is practiced. The consequences for nursing practice are manifold and are based on various factors as described through the identified facilitators and barriers. Future concepts for rapid orientation of agency nursing staff should take into account aspects concerning staff interactions, welcome culture and organisational factors like the appointing of contact persons. Concepts should consider both perspectives of permanent and agency nursing staff. Permanent staff might be supported in the orientation of their temporary colleagues by written protocols and manuals, while shift and ward managers should reflect on their leadership role and the culture they model. Both sides could profit from systematic feedback mechanisms in order to direct personal and organisational development. Induction and rapid orientation concepts should be systematically developed, implemented and evaluated to broaden the limited evidence currently available in this field. On the one hand evidence‐based recommendations may be a helpful guidance for hospitals, while tailored concepts are needed for specialised units, as our study makes tangible the difference between medical specialties, demoting an experienced nurse in one area to a novice in a different area. Further research should also focus on the analysis and change management of individual ward and leadership cultures as a relevant contextual factor in the implementation of induction concepts.

## 6 Conclusion

This meta‐synthesis has yielded a comprehensive overview of the experiences of agency nursing staff in the hospital setting related to induction and rapid orientation to new wards, and the corresponding facilitators and barriers. Our findings highlight the sometimes difficult relationship between agency and permanent nurses and their challenging collaboration, which might benefit from increased mutual sensitivity and empathy. Since agency nursing staff is part of the reality of nursing practice, the goal should be to ensure the quality of the work of agency nurses and to relieve the permanent nurses of the burden of induction as much as possible. In this respect, concepts are needed with which hospitals could develop their own procedures, and which are developed and assessed in methodologically rigorous studies.

## Author Contributions

J.T.: writing – original draft, conceptualization, methodology, formal analysis, investigation, visualisation. A.B.: writing – review and editing, supervision, conceptualization, methodology, validation.

## Ethics Statement

The authors have nothing to report.

## Conflicts of Interest

The authors declare no conflicts of interest.

## Peer Review

The peer review history for this article is available at https://www.webofscience.com/api/gateway/wos/peer‐review/10.1111/jan.16840.

## Supporting information


File S1.

File S2.

File S3.

Data S1.

## Data Availability

The data that support the findings of this study are openly available and have been attached in the form of supplementary files.
