# Supplementary material for: Induction and Rapid Orientation of Agency Nursing Staff in the Hospital Setting: A Systematic Synthesis of Qualitative Studies
Source: J Adv Nurs. 2025 Feb 27;81(8):5112–29. doi: 10.1111/jan.16840 (PMC12271674; doi:10.1111/jan.16840)
Supplement: Supplementary file 1 — File S1. [file JAN-81-5112-s003.pdf]

Supplementary file 1: Search strategies, list of excluded full texts, methodological quality assessment of included studies

Search strategies

Search of MEDLINE via PubMed

#	Term or strategy	Hits
1	((supplement* [Title/Abstract]) OR (agenc* [Title/Abstract]) OR (casual [Title/Abstract]) OR (bank [Title/Abstract]) OR (temporary [Title/Abstract]) OR (external [Title/Abstract]) OR (contract [Title/Abstract]) OR (travel [Title/Abstract]) OR (flexible [Title/Abstract]) OR (registry [Title/Abstract]) OR (per diem [Title/Abstract]) OR (casualization [Title/Abstract]) OR (non-permanent [Title/Abstract]) OR (outsourc* [Title/Abstract]) OR (float* [Title/Abstract]) OR (relief [Title/Abstract]) OR (locum [Title/Abstract]))	1,277,483
2	((nursing staff [MeSH Terms]) OR (nurs* [Title]))	303,045
3	((acute care [Title/Abstract]) OR (clinic* [Title/Abstract]) OR (hospital*[Title/Abstract]) OR (infirmar*[Title/Abstract]) OR (inpatient* [Title/Abstract]) OR (medical care center* [Title/Abstract]) OR (medical center* [Title/Abstract]) OR (station* [Title/Abstract]) OR (ward* [Title/Abstract]) OR (hospitals [MeSH Terms]) OR (hospital units [MeSH Terms]) OR (hospitalization [MeSH Terms]) OR (inpatients [MeSH Terms]))	5,817,301
4	((qualitat* [Title/Abstract]) OR (interview* [Title/Abstract]) OR (grounded Theor* [Title/Abstract]) OR (focus group* [Title/Abstract]) OR (empirical research [Title/Abstract]) OR (qualitative research [MH]) OR (interview [MH]) OR (focus groups [MH]) OR (grounded theory [MH]) OR (empirical research [MH]))	655,267
5	#1 AND #2 AND #3 AND #4	961
6	#5 using the filter Language: German/ English	918
**Database search completed: July 2021**		
**Update completed: April 2024**		297

Search of CINAHL via EBSCOhost

#	Term or strategy	Hits
1	TI supplemental* OR AB supplemental* OR TI agenc* OR AB agenc* OR TI casual* OR AB casual* OR TI bank OR AB bank OR TI temporary OR AB temporary OR TI external OR AB external OR TI contract OR AB contract OR TI travel OR AB travel OR TI flexible OR AB flexible OR TI registry OR AB registry OR TI "per diem" OR AB "per diem" OR TI casualization OR AB casualization OR TI "non-permanent" OR AB "non-permanent" OR TI outsourc* OR AB outsourc* OR TI float* OR AB float* OR TI relief OR AB relief OR TI locum OR AB locum OR MH "placement agencies+" OR MH "registry personnel+"	256,145
2	TI nurs* OR MH nurses+	469,568
3	TI "acute care" OR AB "acute care" OR TI clinic* OR AB clinic* OR TI hospital* OR AB hospital* OR TI infirmar* OR AB infirmar* OR TI inpatient# OR AB inpatient# OR TI "medical care center#" OR AB "medical care center#" OR TI "medical center#" OR AB "medical center#" OR TI station* OR AB station* OR TI ward# OR AB ward# OR MH hospitals+ OR MH hospital units+ OR MH hospitalization+ OR MH inpatients+	656,225
4	TI qualitat* OR AB qualitat* OR TI interview* OR AB interview* OR TI grounded theor* OR AB grounded theor* OR TI focus group* OR AB focus group* OR TI "empirical research" OR AB "empirical research" OR MH "qualitative studies" OR MH "focus groups" OR MH interviews OR MH "grounded theory" OR MH "empirical research"	419,725
5	S1 AND S2 AND S3 AND S4	1,359
6	S5 using the filter: exclude Medline records	839
**Database search completed: July 2021**		
**Update completed: April 2024**		273

Excluded full texts with reasoning (n = 48)

**Author(s), year**	**Title**	**Reason for exclusion**
Ahmed et al. 2023	Floating to intensive care units: nurses' messages for instant action to promote patient safety	Other focus
Aiken et al. 2013	Hospital use of agency-employed supplemental nurses and patient mortality and failure to rescue	Not a qualitative study
Alvarez et al. 2011	Use of outsourced nurses in long-term acute care hospitals: outcomes and leadership preferences	Not a qualitative study
Baumann et al. 2006	Surge capacity and casualization: human resource issues in the post-SARS health system	Other focus
Birmingham et al. 2019	The experiences of the agency registered nurse: an integrative literature review	Not a qualitative study
Bliss & Alsdorf 1992	Generic orientation for agency nurses	Not a qualitative study
Boyer 1979	The use of supplemental nurses: why, where, how?	Not a qualitative study
Bräutigam et al. 2010	Das letzte Mittel? Leiharbeit in der Pflege	Not a qualitative study
Buchan & Thomas 1995	Profiling flexible nursing staff: bank nurses in Scotland	Not a qualitative study
Cicellin et al. 2015	Dealing with resistance<br>in temporary agency nurses. The role of fear in identity-building processes	Other focus
Connelly 1994	A naturalistic study of a nursing orientation program	Not a qualitative study
Crowell-Grimme & Garner 2007	Doing it better: putting research into practice. Creating a guide for float nurses.	Not a qualitative study
Elrod 2016	Perks of being a travel nurse in the EP Lab: interview with Ashley Crilly, RN	Not a qualitative study
Erickson et al. 2023	The impact of a structured onboarding program for newly hired nurse practitioners and physician assistants	Other population
Faller 2010	Predictors of job satisfaction and burnout in travel nurses	Other population
George 2020	Wie wirkt sich Personalleasing auf die Pflegequalität aus?	Not a qualitative study
Goodman-Bacon 2007	Who are temporary nurses?	Not a qualitative study
Greenberg 2000	Where will I go? Displaced nurses relate their experiences	Other population

Author(s), year	Title	Reason for exclusion
Han et al. 2021	Use of temporary primary care providers in federally qualified health centers	Other population
Hansapoor & Haghgoshayie 2020	High levels of temporary nurse staffing increase the hazard of death in acute care hospitals	Not a qualitative study
Hennerby & Joyce 2011	Implementation of a competency assessment tool for agency nurses working in an acute paediatric setting	Not a qualitative study
Hickey et al. 2024	The voice of travel nurses: facilitating effective staffing during pandemic and expansion-related demands in a children's hospital	Other focus
Hurst & Smith 2011	Temporary nursing staff - cost and quality issues	Other focus
Jeffs et al. 2015	Identifying strategies to decrease overtime, absenteeism and agency use: insights from healthcare leaders	Other population
Koffel 2011	Graduate nurses' perception of hospital orientation: a case study	Other population
Kramer 2017	Compassion fatigue among travel nurses	Other population
Larson 2012	Staffing patterns of scheduled unit staff nurses vs. float pool nurses: a pilot study	Not a qualitative study
MacPhee 1999	A mixed-method study of flex nurses' work-related social relationships	Other focus
Manias et al. 2003	Agency nursing work in acute care settings: perceptions of hospital nursing managers and agency nurse providers	Other focus
Massey et al. 2009	Managing the complexity of nurse shortages: a case study of bank and agency staffing in an acute care trust in Wales, UK	Other focus
Matlakala & Botha 2015	Intensive care unit nurse managers’ views regarding nurse staffing in their units in South Africa	Other focus
Mertens et al. 2019	Interprofessional collaboration within fluid teams: community nurses' experiences with palliative home care	Other population
Novak 2005	Agency nurse assessment for competency	Not a qualitative study
Paul 2020	Nurse staffing solution: what to expect from per diem staff	Not a qualitative study
Peerson et al. 2002	Agency nursing in Melbourne, Australia: a telephone survey of hospital and agency managers	Not a qualitative study
Rispel & Moorman 2015	The indirect costs of agency nurses in South Africa: a case study in two public sector hospitals	Not a qualitative study

**Author(s), year**	**Title**	**Reason for exclusion**
Rudy & Sions 2003	Floating: managing a recruitment and retention issue	Not a qualitative study
Schubert 1995	'You're more your own boss': nurses' experiences of agency work	Other focus
Seo & Spetz 2013	Demand for temporary agency nurses and nursing shortages	Not a qualitative study
Sheridan et al. 1982	Using registry nurses: coping with cost and quality issues	Not a qualitative study
Shinners 2016	CNE SERIES. Quality improvement: creating a float pool specialty within a new graduate residency	Not a qualitative study
Simpson & Simpson 2019	What do we know about our agency nurse population? A scoping review	Not a qualitative study
Sorrentino & Simunek 1991	Nurses' perceptions of temporary nursing service agencies	Not a qualitative study
Stiehl 2004	Quality assurance requirements for contract/agency nurses	Not a qualitative study
Strzalka & Havens 1996	Nursing care quality: comparison of unit-hired, hospital float pool, and agency nurses	Not a qualitative study
Tuttas 2013	Travel nurse job performance: Integration factors as predictors, and travel nurse integration experiences	Other population
Wu & Lee 2006	A comparison study of nursing care quality in different working status nursing staffs: an example of one local hospital	Other focus
Ziebert et al. 2016	Lessons learned: newly hired nurses' perspectives on transition into practice	Other population

Methodological quality assessment of included studies

Question	Berg Jansson & Engström 2017	Collier 2011	FitzGerald et al. 2007	Hass et al. 2006	Krebs et al. 2020	Manias et al. 2003	Muller 2014	Ronnie 2020
**Purpose or research question**								
Was there a clear statement of the aims of the research? [1]	yes	yes	yes	yes	yes	yes	yes	yes
**Study sample**								
Was the recruitment strategy appropriate to the aims of the research? [1]	yes	yes	yes	yes	unclear	yes	yes	unclear
Was there a rationale for the sampling method? [2]	yes	yes	yes	yes	no	yes	yes	yes
Was the sample adequately described? [2]	yes	yes	no	no	unclear	yes	yes	yes
**Data collection**								
Was the data collected in a way that addressed the research issue? [1]	yes	yes	yes	yes	yes	yes	yes	no
Were the methods of data collection described in a way that would allow the study to be repeated? Are the methods transparent and appropriate? [3]	yes	yes	no	yes	yes	yes	unclear	yes
**Data analysis**								
Was the data analysis sufficiently rigorous? [1]	unclear	unclear	no	yes	yes	unclear	unclear	unclear
Was the data analysis approach appropriate for the methodology used? Are the analytical steps transparent? Were "quality control" measures taken? [3]	yes	yes	unclear	yes	yes	yes	yes	yes

[1] Items adapted from the Critical Appraisal Skills Programme (CASP) qualitative studies checklist (www.casp-uk.net)
[2] Items modified from the Consolidated Criteria for Reporting Qualitative Research (COREQ) checklist (Tong et al. 2007)
[3] Items adapted from the Oxford Centre for Evidence-Based Medicine (CEBM) critical appraisal of qualitative studies sheet (www.cebm.ox.ac.uk)
