# Supplementary material for: Induction and Rapid Orientation of Agency Nursing Staff in the Hospital Setting: A Systematic Synthesis of Qualitative Studies
Source: J Adv Nurs. 2025 Feb 27;81(8):5112–29. doi: 10.1111/jan.16840 (PMC12271674; doi:10.1111/jan.16840)
Supplement: Supplementary file 2 — File S2. [file JAN-81-5112-s002.pdf]

Supplementary file 2: Assessment of credibility

Author(s), year	Finding	Supporting verbatim participant quote from the study	Credibility assessment		
			Unequivocal	Credible	Unsupported
Berg Jansson & Engström, 2017	Although the regular CCNs emphasised the necessity to have colleagues to talk with, especially after demanding and ethically challenging situations and mentioned colleagues as a reason to work as regular staff, the agency CCNs did not describe any colleagues as really close to them.	‘‘Agency nurses do not always have the help of colleagues, instead they are more alone.’’ (CCN 2, agency)	X		
Berg Jansson & Engström, 2017	Some of the regular CCNs also mentioned that it sometimes could be draining to constantly meet and introduce new colleagues as a result of an increased level of temporary staffing. The regular CCNs stated that work was more manageable if more regular CCNs or longer term agency CCNs were on duty.	‘‘Since we have patients that are really critically ill, then it’s reassuring to know that I know what colleagues are with me, and who can do what. It’s not that I don’t want to be with new CCNs, but you know what it’s like, I think it’s a bit stressful [to deal with temporary CCNs].’’ (CCN 8, regular)	X		
Berg Jansson & Engström, 2017	No matter what the situation, most agreed that a good team required good communication and leadership.			X	
Berg Jansson & Engström, 2017	They [agency nurses] said that written and clear routines facilitate their work.			X	
Berg Jansson & Engström, 2017	… [agency nurses said] that these instructions [written routines] have to be developed by regular staff.			X	

Author(s), year	Finding	Supporting verbatim participant quote from the study	Credibility assessment		
			Unequivocal	Credible	Unsupported
Berg Jansson & Engström, 2017	The agency CCNs said that the regular CCNs were necessary because they know where to find different rooms and materials.			X	
Berg Jansson & Engström, 2017	Another positive aspect of temporary staffing is illustrated by the regular nurses' description and experience of how this phenomenon has improved routine instructions and quality of documents since this is a prerequisite for the introduction of temporary staff.			X	
Berg Jansson & Engström, 2017	Clear documentation is needed that quickly and clearly provides the temporary CCNs with the information.			X	
Collier, 2011	Participants reported a lack of support from the permanent staff. This lack of support is especially prevalent when the units are very busy and the workload is high despite the usage of agency nurses. During the busy periods, permanent staff would ignore the agency nurses as explained by the following participant:	"There is no support but there is a reason for it, I can't say they do not support but they are so overworked and burnt out, I think that they [permanent staff] got third degree burn out." (Participant 9). "You don't get much support from your colleagues [permanent staff]. You don't have a team spirit" (Participant 8).	X		
Collier, 2011	… Agency nurses depend on the support from permanently employed staff.	"They [permanent staff] are quite accommodating when you come in as an agency nurse and they help you to adapt to your shift because they would like to have you there, that's wonderful" (Participant 3).	X		

Author(s), year	Finding	Supporting verbatim participant quote from the study	Credibility assessment		
			Unequivocal	Credible	Unsupported
Collier, 2011	Orientation is of importance when placing agency nurses in a new environment. (…) This particular participant did not receive adequate support in a new environment and neither was there an available orientation package that could be used.	"I don't think so, I don't think there is enough support when you start in a unit, on that day they [permanent staff] will orientate you around but sometimes you start in a unit that is so busy there is no time for orientation and they give you your patient and you have to ask around where is this and where is that [equipment]" (Participant 7).	X		
Collier, 2011	The lack of support in terms of orientation of new agency nurses could result in poor patient care. Patient concerns that need to be addressed such as emergencies (resuscitation), assessment of patients, family support and so on may go unnoticed.			X	
Collier, 2011	The lack of response or support leads to feelings of anxiety and vulnerability as mentioned by this participant.	"If you look at a new agency nurse, somebody that comes into a new situation, how vulnerable they feel and the unit is not known, they don't know the staff. Now they don't actually know where they stand and then you find that person is vulnerable because where is the support you want. [...] orientation is very important and I come back to that, it's very important" (Participant 4).	X		

Author(s), year	Finding	Supporting verbatim participant quote from the study	Credibility assessment		
			Unequivocal	Credible	Unsupported
Collier, 2011	On the other hand, different opinions exist in terms of the amount of support that is rendered to agency nurses in the clinical setting. Some participants felt that permanent nurses showed support if the agency nurse had worked most of their shifts in one particular unit. Having worked in one unit for most of their shifts allows for true collaboration by both the agency nurse and the permanent staff.	"There is a lot of support ...they [permanent staff] ask for our inputs, they respect us and they get inputs from us about changes in the system, things like that we give input about our problems,....the night manager gives us a lot of support and the staff working with us and yes the doctors too" (Participant 10).	X		
Collier, 2011	… One participant commented that when there is inadequate support at the clinical settings, the onus should lie with the agency nurse to request such help	"I think if I ask for support, it will be there ... and I don't think that we must have that attitude that we are agency-nurses they [permanent staff] must help us all the time. We need to take responsibility and say: I need help now" (Participant 1).	X		
Collier, 2011	Furthermore, fear of reprisal prevents agency nurses to discuss a too heavy workload with the permanent staff. The fear of not being booked to work again is shared by some participants, as at these particular healthcare institutions agency nurses are required on a more regular basis, whilst other healthcare institutions are known for their cancellations.	"Yes, it happens at places that people [agency nurses] are not always communicating. They are scared that they will not be used again' (Participant 4).	X		
FitzGerald et al., 2007	Support from the permanent nurses who have local knowledge can make the nurses more comfortable. [They need] attitudes from the staff that make them feel welcome and safe to ask questions.	... a bit stressful at first but it hasn't been as bad as what I was thinking it would be because the staff are usually helpful and you feel like you can ask if you're not sure. (4:202)	X		
FitzGerald et al., 2007	Agency or non-permanent staff are often left out of the decision-making process.				X

Author(s), year	Finding	Supporting verbatim participant quote from the study	Credibility assessment		
			Unequivocal	Credible	Unsupported
FitzGerald et al., 2007	The nurses in the casual pool are clearly in a prime position to assess the ability of clinical teams to provide this type of context [in which agency staff feels safe to function well] in which nurses from the casual pool can work comfortably.			X	
FitzGerald et al., 2007	They [casual pool nurses] sometimes wonder if they have missed things that they did not know about.			X	
FitzGerald et al., 2007	It is also up to the casual nurse to take an initiative.	… go to the ward and only take the first step to mix in and they're usually more than happy to accept you. (5:168)	X		
FitzGerald et al., 2007	It is felt there is little attempt to allocate patients to casual nurses by matching patient need to the individual casual nurse's ability and experience or indeed what load she might have had the day before. [They need] a fair work allocation that gives them time to work safely.	… they will often say to their staff members that work there, 'who do you want? Which ones do you want?' and it's very obvious and then you just get what's left. (5:178)<br>… but when you have a bad one [shift] it's really really bad and you can be reading sitting in hand over — 'unconscious', 'incontinent', 'confused', 'aggressive' you can tick that's yours, basically you know you're going to get the heaviest of patients. (1:227)<br>… it's not always that good to be a jack of all trades and a master of none, alright yes you can go onto any ward and you can work but your knowledge of that particular area is limited because you can't be an expert at everything. (5:141)	X		

Author(s), year	Finding	Supporting verbatim participant quote from the study	Credibility assessment		
			Unequivocal	Credible	Unsupported
FitzGerald et al., 2007	Unfamiliarity with a place and the patients means that their work often takes longer. Work takes longer because they need to find information and things.	… you just waste so much [time] running around trying to figure out where the medications are, you know, and trying to get into the treatment rooms because they've all got their different codes (1:266)	X		
FitzGerald et al., 2007	Each ward keeps things in different places and finding them takes up time for both the nurse from the casual pool and the permanent staff who have to show them.			X	
FitzGerald et al., 2007	The conventional ward report does not give enough detail about the patient.	… in handover you get down every single patient, a little brief outline of them and nothing specific for your patients. (1:268)	X		
FitzGerald et al., 2007	The nurses like to be given extra information after the handover. Sometimes the senior nurse or another casual will help them by telling them more [about the patients]	… if you had a little bit more in-depth on the patients that you're going to take care of. 'Cause you really don't have time. (1:268)	X		
FitzGerald et al., 2007	… otherwise [if agency nurses aren't given extra patient information], they have to rely on reading the documentation.			X	
FitzGerald et al., 2007	The opportunity to have the same patients more than 1 day in a row does alleviate the need to find information.	… and it's so much more enjoyable the second day and you start to build a relationship with them and you knew exactly when their antibiotics were due without even looking (1:272)	X		
FitzGerald et al., 2007	Some of the [agency] nurses describe a routine that appears to work for them. They find out more information before starting to look after the patients.			X	

Author(s), year	Finding	Supporting verbatim participant quote from the study	Credibility assessment		
			Unequivocal	Credible	Unsupported
FitzGerald et al., 2007	Besides the odd 'thank you' at the end of a shift or flattering invitations to 'come and work with us', they are not given feedback on their performance. Even complaints about their work are vague. A formal system for enabling casual nurses to provide clinical teams with feedback about their performance in the area would be useful for high performing teams in terms of promoting their area and for poorly performing teams to know where they might start to change practices. [Recommendations made as a result of this study are] A formal feedback mechanism for casual nurses regarding their performance	… respect really, we don't get a lot of it, some people on the wards will be [say] thank you for being here, thank you for being able to help us out ... (5:162)	X		
FitzGerald et al., 2007	[The agency nurses need] clear instructions regarding ward policies and procedures.			X	
FitzGerald et al., 2007	[Recommendations made as a result of this study are] A formal feedback mechanism for clinical teams regarding their ability to support casual nurses.				X
Hass et al., 2006	The experience of lacking confidence was described through field text as agency nurses' difficulties with multiple workplaces. Inherent in this are the issues of different technology in ICU's and differing approaches to care, both of which contributed to participants' descriptions of insecurity and diminished self-assurance.			X	

Author(s), year	Finding	Supporting verbatim participant quote from the study	Credibility assessment		
			Unequivocal	Credible	Unsupported
Hass et al., 2006	A major barrier to giving prompt and effective patient care, and to fitting in with the individual intensive care environments, was identified by the participants in the study as utilizing the wide array of different equipment available in the various London ICUs. The participants indicated that if they felt comfortable with the equipment used when they were employed it made their shift run considerably more smoothly. Another advantage was that they required less assistance by the permanent staff members during their shift.	''I think that if you arrive at a place and you are familiar with the bed area and the equipment, like the defib and the ventilators, and even the machines like the IMEDS and that, I think if you feel confident then you can get on with it (the shift).'' (Participant 6)	X		
Hass et al., 2006	Participants in the study explained that it could be a difficult experience to go to a new unit where they might not be familiar with the equipment and/or know where it was stored.	''I need to know where certain things are so that my patient is safe and that is, you know, the arrest trolley and where I can do gases and where the suction equipment is, oxygen [...] and then I'm happy.'' (Participant 2)	X		
Hass et al., 2006	… they described feeling as though their skills were being lost and they felt incompetent in having to ask the permanent staff how to undertake simple tasks.			X	
Hass et al., 2006	The participants stressed the need for self confidence when going to new units and being faced with the possibility of unfamiliar equipment.			X	
Hass et al., 2006	The participants stated that those units that had protocols in use and 'easily on hand' eased their transition into unfamiliar units.	''Good hard information at your fingertips is what you need when you can't come away from the bedside, and when you are new or when you are an agency nurse.'' (Participant 2)	X		

Author(s), year	Finding	Supporting verbatim participant quote from the study	Credibility assessment		
			Unequivocal	Credible	Unsupported
Hass et al., 2006	Protocols that were deemed especially helpful were those addressing pharmacy issues such as concentrations of inotropes and enteral feeding regimes.			X	
Hass et al., 2006	It was felt that 'protocolisation' of a unit aided safety and was helpful in ensuring that the required standard of care could be more efficiently achieved.			X	
Hass et al., 2006	The common sentiment was that participants wished to see all units use protocols or reference folders.			X	
Hass et al., 2006	All participants identified that when working at a unit for the first time the benefits of a unit tour were invaluable.			X	
Hass et al., 2006	Participants explained that they often felt as though they were 'disturbing' other staff members when they did not know where to find equipment and dressings.	''It is tough on your first day to walk into a new unit and say, can you show me around? to someone who looks like they are really busy ... you know, so sometimes I will avoid that particular stress by arriving half of an hour early and just go and have a look around and sort of orientate myself as to where things are and that way when I start work I have an idea where most things are.'' (Participant 1)	X		
Hass et al., 2006	It was felt that hospitals that did not use large numbers of agency nurses were less geared to an effective orientation of the unit, and it was most often in those units where the participants were not given a tour at the beginning of the shift.			X	
Hass et al., 2006	Participants explained that units that regularly used large numbers of agency nurses gave the most effective orientation.			X	

Author(s), year	Finding	Supporting verbatim participant quote from the study	Credibility assessment		
			Unequivocal	Credible	Unsupported
Hass et al., 2006	Participants shared that once they were familiar with the environment and with the equipment, they took considerably less time to undertake routine aspects of care.			X	
Hass et al., 2006	The use of protocols in the intensive care environment allows agency nurses to refer to a unit-recognised document, on which they may base their rationale for care provision, thus improving their experience and delivery of care.			X	
Hass et al., 2006	It is suggested that agency nurses could consider requesting to work in a limited number of ICU's as opposed to numerous units. Agencies themselves could easily facilitate this intervention and help to match the individual agency nurse's experience with the differing case mix found in each ICU.				X
Hass et al., 2006	The hospitals can assist the integration of agency nurses into their environment by ensuring sufficient effective orientation is provided. The use of information sheets about the unit may help to integrate the agency nurses into the unknown environment, and help to improve their familiarity and clinical productivity.			X	
Hass et al., 2006	Key staff members could be made known to the agency nurses at the beginning of the shift to whom the agency nurses could refer questions if the shift manager is busy. This may assist the agency nurse with some necessary clinical support, and ensure the agency nurse's questions and clinical concerns are addressed promptly.			X	

Author(s), year	Finding	Supporting verbatim participant quote from the study	Credibility assessment		
			Unequivocal	Credible	Unsupported
Hass et al., 2006	It is also proposed that introduction of a tour and suitable orientation to the intensive care unit for new agency nurses early in the shift would improve their familiarity with the ICU and perhaps increase their clinical productivity.			X	
Hass et al., 2006	Receiving feedback is a method of improving practice.	''It is great to get feedback as that is the only way you can improve. If I am better at what I am doing then my patient is going to benefit at the end of the day. It doesn't matter if it is positive or negative, it is still feedback.'' (Participant 4)	X		
Hass et al., 2006	The non-existence of receiving honest feedback when working full time as an agency nurse was also discussed.	''Feedback really is an essential part, but not getting feedback is just part and parcel of being an agency nurse.'' (Participant 4)	X		
Krebs et al., 2020	Besonders Einsätze in Nachtdiensten, in denen eine externe Kraft ohne weitere interne Pflegekraft auf einer bestimmten Station im Dienst sei, wurde äußerst kritisch angesehen. Dies lag darin bedingt, dass zum derzeitigen Zeitpunkt, in vielen Fällen Nachtdienste nur mit einer examinierten Pflegekraft besetzt waren. Es wurde befürchtet, die hohe Anzahl der Patient/innen sowie eine geringe Einarbeitung und fehlende Routine der externen Pflegekraft sowie fehlendes Wissen über *„die Räumlichkeiten sowie die Ausstattung aber auch Abläufe und Strukturen“* könnte dazu führen, dass eine qualitativ hochwertige Betreuung von Patient/innen nicht mehr gewährleistet werden könne.	„die Räumlichkeiten sowie die Ausstattung aber auch Abläufe und Strukturen“		X	

Author(s), year	Finding	Supporting verbatim participant quote from the study	Credibility assessment		
			Unequivocal	Credible	Unsupported
Krebs et al., 2020	Es wurde explizit darauf hingewiesen, dass Stationen und Krankenhäuser nicht nach einem übergreifenden Standard eingerichtet bzw. Abläufe und Prozessstrukturen aufgesetzt seien. Auch die Anforderungen auf den verschiedenen Stationen seien z. T. sehr spezifisch.	„das kann ein hochspezialisierter und hoch kompetenter Kollege sein, aber es kann nicht erwartet werden, dass dieser von drei verschiedenen Krankenhäusern, alle spezifischen Abläufe kennt“.	X		
Krebs et al., 2020	Eine routinierte Arbeitsweise mit Kenntnissen über Abläufe, Materiallager, usw. würde sich so nur verzögert und meist in weniger gut ausgeprägter Form gegenüber dem internen Stammpersonal ausprägen.			X	
Krebs et al., 2020	Zusammenfassend wurde der Wirkungsgrad von Leiharbeit als sehr gering eingeschätzt, da Fremdpersonal die *„Situation vor Ort nicht so gut kennen“* und *„eine Menge Geld, für Mitarbeiter ausgegeben [wurde], die nicht voll einsetzbar [waren]“.*	„Situation vor Ort nicht so gut kennen“<br>„eine Menge Geld, für Mitarbeiter ausgegeben [wurde], die nicht voll einsetzbar [waren]“	X		
Krebs et al., 2020	Es wurde jedoch auch angeführt, dass mit der Zeit eine Verbesserung in der Zusammenarbeit mit externen Zeitarbeitsfirmen hergestellt werden konnte z.B. durch die Bitte häufiger dieselben und besser qualifizierte Pflegekräfte auf eine spezielle Station zu senden.			X	
Krebs et al., 2020	Aus den geführten Interviews war ersichtlich, dass Stationspflegefachpersonen gerne dazu bereit seien Wissen mit gut ausgebildeten externen Mitarbeitenden zu teilen.			X	

Author(s), year	Finding	Supporting verbatim participant quote from the study	Credibility assessment		
			Unequivocal	Credible	Unsupported
Krebs et al., 2020	Entstand jedoch das Gefühl, dass das Fremdpersonal über wenig Wissen und Kompetenz verfüge, wurde versucht dieses Personal auszusortieren. Die Beurteilung der entsendeten Personen erfolgte innerhalb weniger Stunden. Entweder die Stationsleitungen erhielten sofort die Rückmeldung, dass das Stationsteam eine Zusammenarbeit mit einer bestimmten externen Pflegekraft nicht wiederhohlen wolle oder die Zusammenarbeit entwickelte sich positiv.			X	
Krebs et al., 2020	Ergänzend muss an dieser Stelle jedoch erläutert werden, dass sich die Erwartungen der Projektpartner stark im Anspruch unterschieden, welche Aufgaben unter einer *„selbstständigen Übernahme"* bestimmter Patientenzimmer verstanden wurde.	„selbstständigen Übernahme"		X	
Krebs et al., 2020	Einerseits wurde erwartet, dass Aufgaben am Patienten bzw. Tätigkeiten der Grundpflege übernommen werden konnten. In diesem Zusammenhang wurde auch Verständnis dafür geäußert, das selbst gut ausgebildetes Fremdpersonal verschiedene Eigenarten differenzierter Stationen und Krankenhäuser nicht auf Anhieb kennen können.			X	
Krebs et al., 2020	Ebenfalls sei es v.a. für Leiharbeitskräfte nicht einfach den Überblick über Prozesse sowie den Aufbau eines spezifischen Krankenhauses zu erkennen.			X	
Krebs et al., 2020	Außerdem wurde deutlich auf ein erschwertes Einbinden von Fremdpersonal in den Pflegealltag einer Station verwiesen.			X	

Author(s), year	Finding	Supporting verbatim participant quote from the study	Credibility assessment		
			Unequivocal	Credible	Unsupported
Krebs et al., 2020	Andererseits wurden Annahmen [der Erwartungen an Fremdpersonal] beschrieben, welche die direkte Pflegetätigkeit übertrafen, sei es Röntgenanmeldungen vorzunehmen oder Laborergebnisse anzufordern usw.			X	
Krebs et al., 2020	Eine Berufsgruppe verwies in diesem Zusammenhang auf ein „allgemeines Sicherheitsrisiko“ hinsichtlich des Einsatzes von fremden Pflegekräften, welches sie scheuen würde. Dieses wurde damit begründet, dass externes Personal nicht so gut eingeschätzt werden könne, wie das eigene Team.	„allgemeines Sicherheitsrisiko“		X	
Krebs et al., 2020	… Einarbeitungsphasen [konnten] zum Zeitpunkt der Interviewführung nur in stark verkürzter Zeit und Form oder *„gar nicht mehr“* erfolgen.	„gar nicht mehr“		X	
Krebs et al., 2020	Gleichzeitig wurde angemerkt, dass Fremdpersonal als Entlastung eingesetzt werden sollte, diese sich jedoch nicht einstelle bzw. nicht von den Stationskräften wahrgenommen wurde, wenn der externen Pflegekraft zunächst alles gezeigt werden musste.	„Also ich denke mal, da haben alle Beteiligten ein Interesse dran, also dass das also vernünftig läuft, aber die Grenzen, die es halt gibt, die sind halt der Situation geschuldet, ne und wenn man halt nur zwei oder drei Tage da ist, also, da kann man sich also nicht den ganzen Tag daneben stellen“.	X		
Krebs et al., 2020	So wurde beschrieben, dass es zum einen Schichtleitungen gäbe, die eine externe Pflegekraft freundlich begrüße und sofort die wichtigsten Materialien und Räumlichkeiten zeige bzw. sich über *„zwei weitere Hände freut“*. Auf der anderen Seite wurde eine Leitung charakterisiert, *„bei der sich das Gegenüber schon nach der Begrüßung gerne wieder verabschieden würde“.*	„zwei weitere Hände freut“<br><br>„bei der sich das Gegenüber schon nach der Begrüßung gerne wieder verabschieden würde“.	X		

Author(s), year	Finding	Supporting verbatim participant quote from the study	Credibility assessment		
			Unequivocal	Credible	Unsupported
Krebs et al., 2020	Dem gegenüber wurde ebenfalls berufsgruppenübergreifend die Wahrnehmung kommuniziert, dass einrichtungsintern kein einheitliches Einarbeitungskonzept existieren würde.			X	
Krebs et al., 2020	Ebenfalls wurde illustriert, dass auf Stationen keine Zeit bliebe im Dienst Mitarbeitende einzuarbeiten, die als vollwertige Planstelle im Dienstplan eingeplant seien.			X	
Krebs et al., 2020	Neben Kosten, die für eine Station entstehen, wenn eine Pflegekraft im Dienst ist, die nicht alle Aufgaben vollwertig übernehmen kann wurden verschiedene praktische Alltagssituation beschrieben, welche in vielen Bereichen keine strukturierte, zeitlich ausreichende und fachgerechte Einarbeitung ermöglichte. Unter anderem wurde der spezifische Fall einer neuen fest eingestellten Pflegekraft beschrieben, in welchem Zusammenhang sich genau an einem Tag die Möglichkeit ergeben hatte, eine zusätzliche Pflegekraft im Dienst einsetzen zu können, sodass Ansätze einer Einarbeitung erfolgen konnten.			X	
Krebs et al., 2020	An den übrigen Tagen war die betroffene neue Pflegekraft auf sich alleine gestellt und musste sich durch gezielte Fragen, Lesen, Beobachten oder Ähnlichem alles Weitere selbst beibringen.			X	
Krebs et al., 2020	Generell wurden Geräteinweisungen zwar durchgeführt, allerdings nicht immer von dafür ausgebildeten Pflegekräften, sondern von denjenigen, die kurzfristig zur Verfügung standen.			X	

Author(s), year	Finding	Supporting verbatim participant quote from the study	Credibility assessment		
			Unequivocal	Credible	Unsupported
Krebs et al., 2020	Aufgrund der fehlenden Einarbeitung, wurde sich zum Zeitpunkt der Interviewführung mit Stationsmappen, die Kurzanweisungen oder Flussdiagramme enthielten beholfen, um es stationsfremden Mitarbeitenden zu ermöglichen, selbstständig einfache Abläufe zu erschließen und durchzuführen.			X	
Krebs et al., 2020	Zusammenfassend wurde vor einer fehlenden Einarbeitung in Zukunft gewarnt, da Fremdpersonal auf eine Station gerufen werde, wo bereits eine hohe Belastung vorliege und erwartet würde, dass die externe Unterstützung schnell und selbstständig Aufgaben übernehmen könne.	„Und es fängt ja schon an, wie ist hier unser Notrufsignal. Also wie hole ich jemandem, wenn Reanimationsfall ist und so weiter. Geht da was schief, dann haben wir ein Problem und zwar ein richtiges, ja. Ist ein Organisationsverschulden, weil sie Leute eingesetzt haben, die nicht wissen, wie es funktioniert“.	X		
Krebs et al., 2020	Ebenso sollte [von Agency Nurses] Hilfe angeboten werden, engagiert und motiviert Aufgaben erfüllt werden und ein freundlicher Zugang auf Teammitglieder sowie auf die Patient/innen der Station gezeigt werden.	„gewissen Ausstrahlung“<br>„gewissen Standing“		X	
Krebs et al., 2020	Als sehr wichtig wurde es eingestuft, dass Fachkräfte, die in mehreren Krankenhäusern eingesetzt werden, an jedem Einsatzort eine gute Einarbeitung erhielten; besonders auf den Stationen, die oft Bedarfsanforderungen stellten. Es wurden 3 – 6 Monate pro Träger geäußert, um die jeweiligen „Interna eines Hauses zu durchschauen“ und Kenntnisse über routinierte Prozesse aufzubauen,			X	

Author(s), year	Finding	Supporting verbatim participant quote from the study	Credibility assessment		
			Unequivocal	Credible	Unsupported
Krebs et al., 2020	[Ausreichende Einarbeitung wurde] als Grundlage angesehen […], die seitens des Stammpersonals als störend empfundenen *„permanenten Fragen“* zu verringern.	„permanenten Fragen“	X		
Krebs et al., 2020	Im optimalen Fall sollte eine Einarbeitung entsprechend der Vorerfahrungen der neuen Mitarbeitenden durch eine Ansprechperson auf der jeweiligen Station erfolgen.			X	
Krebs et al., 2020	Außerdem sollte bei einer zentral organisierten Einarbeitung die Fachbereiche der letzten Arbeitseinsätze der neuen Pflegekraft berücksichtigt werden, sodass nach Möglichkeit sowohl ein Einsatz im internistischen aber auch chirurgischen Fachbereich ermöglicht würde.				X
Krebs et al., 2020	Darüber hinaus könnte die Einarbeitungszeit dafür genutzt werden, ein erstes Feedback über die Einsatzfähigkeit dieser Pflegekräfte einzuholen.			X	
Krebs et al., 2020	Dem gegenüber wurde darauf verwiesen, dass ein Einarbeitungsbedarf institutsspezifischer Organisationsstrukturen dadurch verringert werden könnte, dass die hausinterne Schichtleitung so gut auf eine Zusammenarbeit mit einer externen Kraft vorbereitet sei, dass sie Aufgaben zielsicher erklären und anschließend delegieren könne.			X	

Author(s), year	Finding	Supporting verbatim participant quote from the study	Credibility assessment		
			Unequivocal	Credible	Unsupported
Krebs et al., 2020	Ebenso ist es entscheidend im Vorfeld eine Erwartungshaltung zu kreieren, welche seitens des Fremdpersonals auch erfüllt werden kann. Hierfür ist eine Transparenz über Stärken, unterstützendes Potenzial und Kompetenzen des Fremdpersonals sowie dem anfallenden Bedarf auf einer spezifischen Station nötig, sodass bilateral Klarheit hinsichtlich des Erwarteten besteht.			X	
Krebs et al., 2020	Allerdings können zentralisierte Module [der Einarbeitung] auch für Fremdpersonal sinnvoll konzipiert werden. Darüber hinaus besteht die Möglichkeit spezifisches Wissen in E-Learning-Formaten aufzusetzen, sodass verschiedene Lerneinheiten vor dem Einsatz auf einer speziellen Station durchgeführt, wiederholt und ins Gedächtnis gerufen werden können.				X
Manias et al., 2003	Orientation was an important consideration for agency nurses. Participants referred to the orientation provided by their agency and by a particular hospital.			X	
Manias et al., 2003	Nursing agencies prepared nurses for their role by communicating information at interview and by written handouts. Most participants indicated that the agencies provided information about a professional code of conduct, fees, uniforms, and agency regulations for working and cancelling shifts. Only one participant commented that the agency provided comprehensive information about hospitals, including geographical location, car parks, patient system of care, and procedures to follow for emergency situations.	There was a brief statement about the code of conduct but there was no orientation about the hospitals. It was mostly about fees and uniforms.	X		

Author(s), year	Finding	Supporting verbatim participant quote from the study	Credibility assessment		
			Unequivocal	Credible	Unsupported
Manias et al., 2003	Agency nurses received some form of orientation on their first visit to a particular hospital. Thereafter, hospital employees assumed that agency nurses were familiar with an environment if they returned to work there.	Usually you get an orientation the first time you go there [to the hospital]. Some places give you a fabulous orientation so you know where all the fire exits are, and what the emergency procedures are, the people you are responsible to and the codes … Every hospital has a different code … and they don't tell you every time. I was at a hospital that I work at a lot quite recently and they devised an orientation checklist for staff but during my shifts I was lucky to fill out half of it and I have worked at that hospital on and off for six years.	X		
Manias et al., 2003	Furthermore, all agency nurses agreed that the hospital employees should make available a written orientation package for their ward setting. This package could be then taken away for future reference.	In the written package there should be the mission statement of the hospital. I think they should have objectives of what they want the nurse to achieve for the day. They should have something written about the legalities of documentation and a section about nurse registration. They should also have their fire drills and their code for resuscitation. If there are any problems or if there is a lack of support, agency nurses should have the contact names of hospital people they can contact.	X		

Author(s), year	Finding	Supporting verbatim participant quote from the study	Credibility assessment		
			Unequivocal	Credible	Unsupported
Manias et al., 2003	In relation to nursing support, participants believed they received adequate help during their working shift. At times, however, agency nurses did not feel well supported in the clinical area. Interestingly, this situation occurred in settings where they had regularly worked in an environment, and permanent staff perceived that agency nurses were relatively familiar with policies and protocols.	Sometimes the person-in-charge has got their own workload and that is where you get problems with support because there is no one around.	X		
Manias et al., 2003	Participants also felt that they were inadequately supported in extremely busy situations because permanent nurses had exorbitant workloads despite agency allocation.	Sometimes the [permanent] nurses haven't got the time or the resources to have someone around to ask questions. They are just too busy.	X		
Manias et al., 2003	Occasionally permanent nurses ignored participants' requests for help, especially if they involved procedures that disrupted particular ward routines.	On this aged care rehab. [rehabilitation] setting I felt that I was disrupting the nurses' routines. Three staff had gone to dinner so that left me with two other nurses. The two other nurses were going around together putting patients to bed. They were not answering any buzzers — I was answering the buzzers. I would say, 'Can I have some help, this patient needs to go to the toilet.' And they would say, 'Oh no, I am putting these patients to bed.' It was just inappropriate.	X		
Manias et al., 2003	However, aside from refusing to send the agency nurses back to these ward settings, there was no further follow-up with the hospitals.			X	

Author(s), year	Finding	Supporting verbatim participant quote from the study	Credibility assessment		
			Unequivocal	Credible	Unsupported
Manias et al., 2003	Since agency nurses were not present in the setting for a prolonged period, they all experienced a sense of urgency about voicing any concerns with permanent staff.	In the couple of places where I've had really bad experiences, I have told the person-in-charge … by the end of the shift they haven't done anything to help me or resolve the issue, I would usually say to them that I was very disappointed in this and for this reason, and that I won't return to the unit.	X		
Manias et al., 2003	Participants also perceived that communicating about patient care information tended to focus on tasks requiring completion rather than on the holistic needs of patients.	When I ask for information about a patient's past history so I can care for them better, I usually don't get the right information. I usually just get the comment, 'You just have to do this thing,' rather than answer my question so I can make my own decision on what I am going to do. I feel that they don't really let me know the patient's holistic picture because they want me to do a series of tasks.	X		
Manias et al., 2003	This reliance upon self-education and individual responsibility appeared to be related to nurses' perceptions about their knowledge deficits.	I think it's your own responsibility to make sure you are on top of things, and if you are not then to find out or access people who can get you up-to-date. Only you would know what your deficits are.	X		
Manias et al., 2003	For agency nurses, the amount of time available for orientating about patient care needs and the nuances of the environment may be limited. For permanent nurses, they are required to address the additional workload until the agency nurses' arrival.			X	

Author(s), year	Finding	Supporting verbatim participant quote from the study	Credibility assessment		
			Unequivocal	Credible	Unsupported
Manias et al., 2003	Although some participants enjoyed supportive relationships that served to enrich their practice, others described a lack of supportive consultation especially in settings where they worked regularly and in relatively busy situations.			X	
Manias et al., 2003	The findings reinforce the need to create strong collaborative networks between agency nurses, nursing agencies and hospital institutions, in which all stakeholders could discuss issues of concern and negotiate an agreed position.				X
Muller, 2014	The hospital included some of the participants in their hospital induction which took place over five days and the agency were not paid to attend but were encouraged to attend for their own benefit.	… now they go for induction I didn't know that they go for induction but they go for induction but before it was only permanent staff but now they go for induction but ja its good … (Participant 11).	X		
Muller, 2014	There were different opinions regarding orientation and the length of the orientation. One participant felt it was too long and was a form of abuse.	… to me the time that is given is long to say a person must be orientated for 2 days that is how many hours 24 hour here we are talking about a person that is working in ICU nee that will be the orientation of the environment will be the environment the structural environment and then the charts you see because we are not using the same charts and then to me that cannot take 2 days which now when I talk about this thing I think of the extra hours that she is working I take it as an exploitation because one day she is enough because here we are talking about a sister that is permanent that is working in ICU you understand so the orientation is enough … (Participant 3)	X		

Author(s), year	Finding	Supporting verbatim participant quote from the study	Credibility assessment		
			Unequivocal	Credible	Unsupported
Muller, 2014	Other participants felt it [2 day orientation] was enough and it helps them and some expressed that some participants needed more than the 2 days.	… before I go in the ward we do 2 days orientation before we can't go in the ward we can't just go. I can say it was enough but to others it is not enough because others they ask extra days you see like the lady that was staying with me it was supposed to be 2 days she ask the third day … (Participant 11).<br>… It was very good I found some support from the staff they were good were keen to show me everything it was only me I though o my God no it is the first place I been working in this unit if I am going to cope but I had enough support enough support … (Participant 4).	X		
Muller, 2014	The same participant shared that, if the permanent staff were more patient she would have learnt, as she functions well now.	… they have this thing about the agency staff they like we don't know anything you see but we can't do something they we haven't done before you see … (Participant 8)	X		

Author(s), year	Finding	Supporting verbatim participant quote from the study	Credibility assessment		
			Unequivocal	Credible	Unsupported
Muller, 2014	It was suggested that the unit manager's attitude played a very important role in the agency nurse feeling respected and for the teamwork to be effective.	… for the unit to work to function well it is the leader first to respect the agency staff I have seen this if she is not weighing them at the same level the permanent staff think they are supers of the agency staff irrespective of how senior you are to them I am talking to a level of a assistant nurse and a professional nurse you will see that the assistant nurse is not giving you respect you will see that the assistant nurse don't respect you as a sister because of the attitude of the unit manger but if the unit manager respect the staff you will see the team work how it goes we will work like sisters and brothers … (Participant 3)	X		
Ronnie, 2020	… Lack of explanation and poor communication from the ICU manager lay at the heart of the agency nurses' unhappiness.			X	
Ronnie, 2020	A lack of familiarity with the full range of duties undertaken by an ICU nurse, typically performed in conjunction with others, gave rise to feelings of inequality. Perceptions of unfair task allocation were rife.	'When I come to the unit, I get two patients compared to the permanent nurses who just get one. I'm [for]ever tired' (Thato, registered nurse)	X		
Ronnie, 2020	Agency nurses often arrived at their assigned ICUs with minimal to no prior training or orientation and believed they could perform better if they had been exposed to some level of preparation.	'[We are made to feel that] we know absolutely nothing and it's unfair towards others who must take us' (Cecilia, enrolled nurse)<br>'A lot of agency staff are from other parts of the country that have no ICU that provides training. Give us some time to learn' (Betty, enrolled nurse assistant)	X		

Author(s), year	Finding	Supporting verbatim participant quote from the study	Credibility assessment		
			Unequivocal	Credible	Unsupported
Ronnie, 2020	However, the nature of the ICU is that there was often no time for formal training …			X	
Ronnie, 2020	… the ICU nurse was expected to do all the required clinical tasks. Even agency nurses with significant years of experience felt at sea within their allocated ICUs.	'I have past paediatric ICU knowledge, so I have some experience, but I don't have in-depth knowledge of adult ICUs. I'm just starting to build up that knowledge' (Mariam, registered nurse)<br>'They leave me just because I'm an experienced nurse. But I don't know everything in ICU' (Cecilia, enrolled nurse)	X		
Ronnie, 2020	Agency nurses, with a keen sense of their own limitations, had also indicated explicitly to the ICU manager when they felt out of their depth professionally	They allocated me to a very sick patient and I said to the manager ''this is outside my scope of practice. I don't know how to use the machines that they need". Then they took me off that patient' (Elsie, enrolled nurse)	X		
Ronnie, 2020	However, the strategy of simply removing an agency nurse from a patient because she had no prior knowledge of the procedural process to be followed had an unintended negative consequence	'I asked what I must do as I haven't ever taken someone off dialysis. The manager said ''fine, just leave it". So, now I still don't know what to do or where to start' (Thato, registered nurse)	X		

Author(s), year	Finding	Supporting verbatim participant quote from the study	Credibility assessment		
			Unequivocal	Credible	Unsupported
Ronnie, 2020	As agency nurses report directly to the ICU managers after being allocated to the specific ICU, the interactions between these two parties set the tone for what would follow. When asked what role ICU managers should play, agency nurses replied that visibility, availability and on-the-job training were key elements	'He's very patient: he's a top guy. He walked me through the procedure step-by-step. I was very happy. I asked him: ''Can I please, please come back here tomorrow?"' (Hayley, enrolled nurse)<br>'The role of the manager is make sure that everyone is happy when they're working. When staff ask questions, they must be able to explain to them rather than saying ''you're asking too much"' (Noni, enrolled nurse)<br>'They need to be working alongside the nurses. This can help a lot, to be hands-on. If there's new equipment, you need to know how it works. As an agency nurse, I go to the manager to find out as she's the first port of call' (Mariam, registered nurse)<br>'The manager should take me around and explain the status of the patients. She should show me things I'm supposed to know' (Elsie, enrolled nurse)	X		
Ronnie, 2020	… The opportunity to learn and remain motivated was influenced by the workplace culture – as shown in the examples of feedback and support – that was created by ICU managers			X	
Ronnie, 2020	Just as ICU managers often set the tone for the level of collaboration with agency nurses in their ICUs, so the work relationships between agency and permanent nurses typically followed suit.	'The permanents shouldn't look down upon us, like we don't know anything. Talk to us as human beings. Talk to us, tell us what to do. We do have feelings' (Lily, enrolled nurse)	X		

Author(s), year	Finding	Supporting verbatim participant quote from the study	Credibility assessment		
			Unequivocal	Credible	Unsupported
Ronnie, 2020	Positive aspects of the agency nurse experience included feedback and support from permanent staff including ICU managers and instances of belonging and acceptance.			X	

ICU: intensive care unit; CCN: critical care nurse.
