# Supplementary material for: Induction and Rapid Orientation of Agency Nursing Staff in the Hospital Setting: A Systematic Synthesis of Qualitative Studies
Source: J Adv Nurs. 2025 Feb 27;81(8):5112–29. doi: 10.1111/jan.16840 (PMC12271674; doi:10.1111/jan.16840)
Supplement: Supplementary file 3 — File S3. [file JAN-81-5112-s001.pdf]

Supplementary file 3: Process of data aggregation

Four categories resulted exclusively from ‘credible’ findings, which were marked with a superscript ‘a’.

The findings and quotations from Krebs et al. (2020) were translated from German to English language.

Table of contents

Overarching theme 1: Orientation in a new or unfamiliar environment 1

Synthesis 1: Agency nursing staff requires orientation on each new ward in order to do their job. 1

Synthesis 2: Agency nursing staff relies on permanent staff to be prepared for orientation and induction. 3

Synthesis 4: Individualized orientation aided by key staff members may be helpful to agency nursing staff. 7

Overarching theme 2: Working as agency nursing staff 8

Synthesis 5: Agency nursing staff may require patience, time and extra information to care for patients safely. 8

Synthesis 6: Interactions between agency and permanent nurses may be difficult or conflict ridden, depending on support of nursing management. 10

Synthesis 7: The level of support offered by permanent staff can influence how respected or insecure agency nursing staff feels. 13

Synthesis 8: Ever-changing agency nursing staff may pose as a burden on permanent staff. 15

Synthesis 9: The lack of feedback mechanisms for and from agency nursing staff may hinder professional development and collaboration. 16

Synthesis 10: Agency nursing staff needs to be proactive, confident and self-reliant. 18

References 20

Overarching theme 1: Orientation in a new or unfamiliar environment

Synthesis 1: Agency nursing staff requires orientation on each new ward in order to do their job.

Finding	Direct supporting quotation from the study	Category
Orientation is of importance when placing agency nurses in a new environment. (…) This particular participant did not receive adequate support in a new environment and neither was there an available orientation package that could be used. (Collier, 2011)	"I don't think so, I don't think there is enough support when you start in a unit, on that day they [permanent staff] will orientate you around but sometimes you start in a unit that is so busy there is no time for orientation and they give you your patient and you have to ask around where is this and where is that [equipment]" (Participant 7).	Orientation is pertinent to agency nursing staff.
[The agency nurses need] clear instructions regarding ward policies and procedures. (FitzGerald et al., 2007) [a]		
It was rated as very important that professionals who are assigned to multiple hospitals, received a good induction at each site, especially on the wards especially which often required agency nurses. It was said that three to six months were needed per hospital to "understand internals" of each hospital and build knowledge of routines. (Krebs et al., 2020) [a]		
Orientation was an important consideration for agency nurses. Participants referred to the orientation provided by their agency and by a particular hospital. (Manias et al., 2003) [a]		
All participants identified that when working at a unit for the first time the benefits of a unit tour were invaluable. (Hass et al., 2006) [a]		
In summary, it was warned against a lack of familiarization in the future, since external personnel are called to a ward where there is already a high workload and it is expected that the external support can quickly take over tasks independently. (Krebs et al., 2020)	"And it already starts with what's our emergency call here. So how do I get someone if there is a resuscitation case and so on. If something goes wrong, then we have a problem and a real one, yes. It's the fault of the organization because they used people who don't know how things work."	

Participants in the study explained that it could be a difficult experience to go to a new unit where they might not be familiar with the equipment and/or know where it was stored. (Hass et al., 2006)	‘‘I need to know where certain things are so that my patient is safe and that is, you know, the arrest trolley and where I can do gases and where the suction equipment is, oxygen [...] and then I’m happy.’’ (Participant 2)	Knowing the equipment plays a role in keeping patients safe.
A major barrier to giving prompt and effective patient care, and to fitting in with the individual intensive care environments, was identified by the participants in the study as utilizing the wide array of different equipment available in the various London ICUs. The participants indicated that if they felt comfortable with the equipment used when they were employed it made their shift run considerably more smoothly. Another advantage was that they required less assistance by the permanent staff members during their shift. (Hass et al., 2006)	‘‘I think that if you arrive at a place and you are familiar with the bed area and the equipment, like the defib and the ventilators, and even the machines like the IMEDS and that, I think if you feel confident then you can get on with it (the shift).’’ (Participant 6)	
It was explicitly pointed out that wards and hospitals were not set up according to an overarching standard or that procedures and process structures were not set up. The requirements on the various wards were also very specific in some cases. (Krebs et al., 2020)	“this may be a highly specialized and highly competent colleague, but he or she cannot be expected to know all the specific procedures of three different hospitals.”	Each ward is different.
Each ward keeps things in different places and finding them takes up time for both the nurse from the casual pool and the permanent staff who have to show them. (FitzGerald et al., 2007) [a]		
It is also not easy, especially for temporary workers, to gain an overview of processes and the structure of a specific hospital. (Krebs et al., 2020) [a]		
Participants shared that once they were familiar with the environment and with the equipment, they took considerably less time to undertake routine aspects of care. (Hass et al., 2006) [a]		Familiarity with the ward can boost efficiency of agency nursing staff.
It is also proposed that introduction of a tour and suitable orientation to the intensive care unit for new agency nurses early in the shift would improve their familiarity with the ICU and perhaps increase their clinical productivity. (Hass et al., 2006) [a]		
Unfamiliarity with a place and the patients means that their work often takes longer. Work takes longer because they need to find information and things. (FitzGerald et al., 2007)	‘’... you just waste so much [time] running around trying to figure out where the medications are, you know, and trying to get into the treatment rooms because they’ve all got their different codes’’ (1:266)	

Synthesis 2: Agency nursing staff relies on permanent staff to be prepared for orientation and induction.

Finding	Direct supporting quotation from the study	Category
… Agency nurses depend on the support from permanently employed staff. (Collier, 2011)	"They [permanent staff] are quite accommodating when you come in as an agency nurse and they help you to adapt to your shift because they would like to have you there, that's wonderful." (Participant 3)	Permanent staff plays an important role in providing orientation for agency nursing staff.
In particular, night duty assignments in which an external nurse was on duty on a particular ward without another internal nurse were viewed extremely critically. This was due to the fact that at the current time, in many cases night services were staffed with only one registered nurse. It was feared that the high number of patients and a low level of familiarization and lack of routine on the part of the external nurse, as well as a lack of knowledge about "the premises and the equipment, but also the processes and structures" could lead to a situation in which the high-quality care of patients could no longer be guaranteed. (Krebs et al., 2020) [a]	"the premises and the equipment, but also the processes and structures"	
… [agency nurses said] that these instructions [written routines] have to be developed by regular staff. (Berg Jansson & Engström, 2017) [a]		
The agency CCNs said that the regular CCNs were necessary because they know where to find different rooms and materials. (Berg Jansson & Engström, 2017) [a]		
In general, although equipment briefings were provided, they were not always provided by nurses trained to do so, but by those who were available on short notice. (Krebs et al., 2020) [a]		
Clear documentation is needed that quickly and clearly provides the temporary CCNs with the information. (Berg Jansson & Engström, 2017)[a]		Clear documentation is a prerequisite for agency nursing work. [a]
Another positive aspect of temporary staffing is illustrated by the regular nurses' description and experience of how this phenomenon has improved routine instructions and quality of documents since this is a prerequisite for the introduction of temporary staff. (Berg Jansson & Engström, 2017) [a]		

Due to the lack of familiarization, at the time of the interview, ward folders containing short instructions or flowcharts were used to enable staff from other wards to independently understand and carry out simple procedures. (Krebs et al., 2020) [a]		
The hospitals can assist the integration of agency nurses into their environment by ensuring sufficient effective orientation is provided. The use of information sheets about the unit may help to integrate the agency nurses into the unknown environment, and help to improve their familiarity and clinical productivity. (Hass et al., 2006) [a]		
Furthermore, all agency nurses agreed that the hospital employees should make available a written orientation package for their ward setting. This package could be then taken away for future reference. (Manias et al., 2003)	''In the written package there should be the mission statement of the hospital. I think they should have objectives of what they want the nurse to achieve for the day. They should have something written about the legalities of documentation and a section about nurse registration. They should also have their fire drills and their code for resuscitation. If there are any problems or if there is a lack of support, agency nurses should have the contact names of hospital people they can contact.''	Written orientation and protocols work as a facilitator of agency nursing work.
The common sentiment was that participants wished to see all units use protocols or reference folders. (Hass et al., 2006) [a]		
Protocols that were deemed especially helpful were those addressing pharmacy issues such as concentrations of inotropes and enteral feeding regimes. (Hass et al., 2006) [a]		
The participants stated that those units that had protocols in use and 'easily on hand' eased their transition into unfamiliar units. (Hass et al., 2006)	''Good hard information at your fingertips is what you need when you can't come away from the bedside, and when you are new or when you are an agency nurse.'' (Participant 2)	
They [agency nurses] said that written and clear routines facilitate their work. (Berg Jansson & Engström, 2017) [a]		
It was felt that 'protocolisation' of a unit aided safety and was helpful in ensuring that the required standard of care could be more efficiently achieved. (Hass et al., 2006) [a]		

The use of protocols in the intensive care environment allows agency nurses to refer to a unit-recognised document, on which they may base their rationale for care provision, thus improving their experience and delivery of care. (Hass et al., 2006) [a]		

Synthesis 3: Time constraints and lack of routines may hinder effective orientation.

Finding	Direct supporting quotation from the study	Category
In addition to the costs incurred by a ward when a nurse is on duty who is not able to take over all tasks in full, various practical everyday situations were described which in many areas did not allow for a structured, sufficient and professional induction. Among other things, the specific case of a new, permanently employed nurse was described, in which exactly on one day the possibility arose to deploy an additional nurse on duty, so that the beginnings of an induction could take place. (Krebs et al., 2020) [a]		Often, there is not sufficient time for orientation.
Participants also felt that they were inadequately supported in extremely busy situations because permanent nurses had exorbitant workloads despite agency allocation. (Manias et al., 2003)	‘’Sometimes the [permanent] nurses haven’t got the time or the resources to have someone around to ask questions. They are just too busy.‘’	
However, the nature of the ICU is that there was often no time for formal training. (Ronnie, 2020) [a]		
For agency nurses, the amount of time available for orientating about patient care needs and the nuances of the environment may be limited. For permanent nurses, they are required to address the additional workload until the agency nurses’ arrival. (Manias et al., 2003) [a]		
It was also illustrated that wards do not have time to train employees on duty who are scheduled as full positions in the shift roster. (Krebs et al., 2020) [a]		
... familiarization phases could only take place in a greatly reduced form or “no longer at all” at the time of the interview. (Krebs et al., 2020) [a]	‘’no longer at all“	

Participants explained that units that regularly used large numbers of agency nurses gave the most effective orientation. (Hass et al., 2006) [a]		Lack of uniform orientation routines may hinder effective induction.
Agency nurses received some form of orientation on their first visit to a particular hospital. Thereafter, hospital employees assumed that agency nurses were familiar with an environment if they returned to work there. (Manias et al., 2003)	''Usually you get an orientation the first time you go there [to the hospital]. Some places give you a fabulous orientation so you know where all the fire exits are, and what the emergency procedures are, the people you are responsible to and the codes […] Every hospital has a different code […] and they don't tell you every time. I was at a hospital that I work at a lot quite recently and they devised an orientation checklist for staff but during my shifts I was lucky to fill out half of it and I have worked at that hospital on and off for six years."	
It was felt that hospitals that did not use large numbers of agency nurses were less geared to an effective orientation of the unit, and it was most often in those units where the participants were not given a tour at the beginning of the shift. (Hass et al., 2006) [a]		
It was described that on the one hand there were shift leaders who greeted an external nurse in a friendly manner and immediately showed the most important materials and rooms or were "happy to have two more hands". On the other hand, a manager was characterized "where the counterpart would already like to say goodbye again after the greeting". (Krebs et al., 2020)	"happy to have two more hands"<br>"where the counterpart would already like to say goodbye again after the greeting"	
On the other hand, the perception was also conveyed that there is no uniform induction concept within the institution. (Krebs et al., 2020) [a]		
Nursing agencies prepared nurses for their role by communicating information at interview and by written handouts. Most participants indicated that the agencies provided information about a professional code of conduct, fees, uniforms, and agency regulations for working and cancelling shifts. Only one participant commented that the agency provided comprehensive information about hospitals, including geographical location, car parks, patient system of care, and procedures to follow for emergency situations. (Manias et al., 2003)	''There was a brief statement about the code of conduct but there was no orientation about the hospitals. It was mostly about fees and uniforms. ''	

Synthesis 4: Individualized orientation aided by key staff members may be helpful to agency nursing staff.

Finding	Direct supporting quotation from the study	Category
On the other hand, it was pointed out that a need for familiarization with institution-specific organizational structures could be reduced if the in-house shift supervisor was so well prepared for working with external staff that he or she could explain tasks unerringly and then delegate them. (Krebs et al., 2020) [a]		Making key staff members known to agency nursing staff may be helpful in orientation. [a]
Key staff members could be made known to the agency nurses at the beginning of the shift to whom the agency nurses could refer questions if the shift manager is busy. This may assist the agency nurse with some necessary clinical support, and ensure the agency nurse's questions and clinical concerns are addressed promptly. (Hass et al., 2006) [a]		
There were different opinions regarding orientation and the length of the orientation. One participant felt it was too long and was a form of abuse. (Muller, 2014)	''to me the time that is given is long to say a person must be orientated for 2 days that is how many hours 24 hour here we are talking about a person that is working in ICU nee that will be the orientation of the environment will be the environment the structural environment and then the charts you see because we are not using the same charts and then to me that cannot take 2 days which now when I talk about this thing I think of the extra hours that she is working I take it as an exploitation because one day she is enough because here we are talking about a sister that is permanent that is working in ICU you understand so the orientation is enough …'' (Participant 3)	Orientation should be flexible to adapt to individual nurse's needs.

Other participants felt it [2 day orientation] was enough and it helps them and some expressed that some participants needed more than the 2 days. (Muller, 2014)	ʻʻ… before I go in the ward we do 2 days orientation before we can't go in the ward we can't just go I can say I can say it is enough but to others to me it was enough but to others it is not enough because others they ask extra days you see like the lady that was staying that was staying with me it was supposed to be 2 days she ask the third day …" (Participant 11).<br>"… It was very good I found some support from the staff they were good were keen to show me everything it was only me I though o my God no it is the first place I been working in this unit if I am going to cope but I had enough support enough support ...ʼʼ(Participant 4).	
Ideally, the new employees should be trained according to their previous experience by a contact person from the respective ward. (Krebs et al., 2020) [a]		
The hospital included some of the participants in their hospital induction which took place over five days and the agency were not paid to attend but were encouraged to attend for their own benefit. (Muller, 2014)	ʻʻ… now they go for induction I didn't know that they go for induction but they go for induction but before it was only permanent staff but now they go for induction but ja its good … ʻʻ (Participant 11)	

Overarching theme 2: Working as agency nursing staff

Synthesis 5: Agency nursing staff may require patience, time and extra information to care for patients safely.

Finding	Direct supporting quotation from the study	Category
The lack of support in terms of orientation of new agency nurses could result in poor patient care. Patient concerns that need to be addressed such as emergencies (resuscitation), assessment of patients, family support and so on may go unnoticed. (Collier, 2011) [a]		Agency nursing staff may not know what they missed in terms of patient care. [a]
They [casual pool nurses] sometimes wonder if they have missed things that they did not know about. (FitzGerald et al., 2007) [a]		

The conventional ward report does not give enough detail about the patient. (FitzGerald et al., 2007)	‘’… in handover you get down every single patient, a little brief outline of them and nothing specific for your patients’’. (1:268)	Detailed patient reports are necessary for agency nursing staff to deliver adequate care.
… otherwise [if agency nurses aren’t given extra patient information], they have to rely on reading the documentation. (FitzGerald et al., 2007) [a]		
Participants also perceived that communicating about patient care information tended to focus on tasks requiring completion rather than on the holistic needs of patients. (Manias et al., 2003)	‘’When I ask for information about a patient’s past history so I can care for them better, I usually don’t get the right information. I usually just get the comment, ‘You just have to do this thing,’ rather than answer my question so I can make my own decision on what I am going to do. I feel that they don’t really let me know the patient’s holistic picture because they want me to do a series of tasks.‘’	
The nurses like to be given extra information after the handover. Sometimes the senior nurse or another casual will help them by telling them more [about the patients]. (FitzGerald et al., 2007)	‘’… if you had a little bit more in-depth on the patients that you’re going to take care of. ‘Cause you really don’t have time.‘’ (1:268)	
However, the strategy of simply removing an agency nurse from a patient because she had no prior knowledge of the procedural process to be followed had an unintended negative consequence. (Ronnie, 2020)	‘’I asked what I must do as I haven’t ever taken someone off dialysis. The manager said ‘’fine, just leave it”. So, now I still don’t know what to do or where to start’’ (Thato, RN).	Agency nursing staff may require extra time to learn new skills.
The same participant shared that, if the permanent staff were more patient she would have learnt, as she functions well now. (Muller, 2014)	‘’… they have this thing about the agency staff they like we don’t know anything you see but we can’t do something they we haven’t done before you see …’’ (Participant 8)	
Agency nurses often arrived at their assigned ICUs with minimal to no prior training or orientation and believed they could perform better if they had been exposed to some level of preparation. (Ronnie, 2020)	‘’[We are made to feel that] we know absolutely nothing and it’s unfair towards others who must take us’’ (Cecilia, enrolled nurse). ‘’A lot of agency staff are from other parts of the country that have no ICU that provides training. Give us some time to learn’’ (Betty, enrolled nurse assistant).	
The opportunity to have the same patients more than 1 day in a row does alleviate the need to find information. (FitzGerald et al., 2007)	… and it’s so much more enjoyable the second day and you start to build a relationship with them and you knew exactly when their antibiotics were due without even looking (1:272)	Agency nursing staff being given a chance to continue their care decreases their need for orientation.

However, it was also mentioned that over time an improvement in the cooperation with external temporary staffing agencies could be established e.g. by asking to send the same and better qualified nurses to a specific ward more often. (Krebs et al., 2020) [a]		

Synthesis 6: Interactions between agency and permanent nurses may be difficult or conflict ridden, depending on support of nursing management.

Finding	Direct supporting quotation from the study	Category
A lack of familiarity with the full range of duties undertaken by an ICU nurse, typically performed in conjunction with others, gave rise to feelings of inequality. Perceptions of unfair task allocation were rife. (Ronnie, 2020)	''When I come to the unit, I get two patients compared to the permanent nurses who just get one. I'm [for]ever tired'' (Thato, RN)	Agency nursing staff may feel that patient allocation by permanent staff is unjust.
It is felt there is little attempt to allocate patients to casual nurses by matching patient need to the individual casual nurse's ability and experience or indeed what load she might have had the day before. [They need] a fair work allocation that gives them time to work safely. (FitzGerald et al., 2007)	''… they will often say to their staff members that work there, 'who do you want? Which ones do you want?' and it's very obvious and then you just get what's left.'' (5:178)<br>''… but when you have a bad one [shift] it's really really bad and you can be reading sitting in hand over — 'unconscious'', 'incontinent'', 'confused', 'aggressive' you can tick that's yours, basically you know you're going to get the heaviest of patients.'' (1:227)<br>… it's not always that good to be a jack of all trades and a master of none, alright yes you can go onto any ward and you can work but your knowledge of that particular area is limited because you can't be an expert at everything. (5:141)	

On the other hand, assumptions [of expectations of outside staff] were described that exceeded direct nursing activity, whether it was making x-ray requests or requesting laboratory results, etc. (Krebs et al., 2020) [a]		
It is also crucial to create an expectation in advance that can be met by the external staff. This requires transparency about the strengths, supportive potential and competencies of the external staff as well as the arising needs on a specific ward, so that there is bilateral clarity regarding what is expected. (Krebs et al., 2020) [a]		
On the one hand, it was expected that tasks on the patient or activities of basic care could be taken over. It was however also expressed that even well-trained external staff may not immediately be familiar with the various characteristics of different wards and hospitals at the drop of a hat. (Krebs et al., 2020) [a]		
In addition, however, it must be explained at this point that the expectations of the project partners differed greatly in terms of what tasks were meant by an "independent takeover" of certain patient rooms. (Krebs et al., 2020) [a]	„independent takeover“	Agency nursing staff is confronted with differing and partially unrealistic expectations.
… the ICU nurse was expected to do all the required clinical tasks. Even agency nurses with significant years of experience felt at sea within their allocated ICUs. (Ronnie, 2020)	‘’I have past paediatric ICU knowledge, so I have some experience, but I don’t have in-depth knowledge of adult ICUs. I’m just starting to build up that knowledge’’ (Mariam, registered nurse).<br>‘’They leave me just because I’m an experienced nurse. But I don’t know everything in ICU’’ (Cecilia, enrolled nurse)	
In this context, one professional group referred to a “general safety risk” with regard to the use of external nursing staff, which they would shy away from. The reason given for this was that external personnel could not be assessed as well as their own team members. (Krebs et al., 2020)[a]	‘’general safety risk“	
… Lack of explanation and poor communication from the ICU manager lay at the heart of the agency nurses’ unhappiness. (Ronnie, 2020) [a]		

As agency nurses report directly to the ICU managers after being allocated to the specific ICU, the interactions between these two parties set the tone for what would follow. When asked what role ICU managers should play, agency nurses replied that visibility, availability and on-the-job training were key elements. (Ronnie, 2020)	‘’He’s very patient: he’s a top guy. He walked me through the procedure step-by-step. I was very happy. I asked him: ‘’Can I please, please come back here tomorrow?”’ (Hayley, enrolled nurse)<br>‘’The role of the manager is make sure that everyone is happy when they’re working. When staff ask questions, they must be able to explain to them rather than saying ‘’you’re asking too much”.’’ (Noni, enrolled nurse).<br>‘’They need to be working alongside the nurses. This can help a lot, to be hands-on. If there’s new equipment, you need to know how it works. As an agency nurse, I go to the manager to find out as she’s the first port of call’’ (Mariam, registered nurse).<br>‘’The manager should take me around and explain the status of the patients. She should show me things I’m supposed to know’’ (Elsie, enrolled nurse).	Shift and ward managers are responsible for creating a constructive workplace culture.
… The opportunity to learn and remain motivated was influenced by the workplace culture – as shown in the examples of feedback and support – that was created by ICU managers. (Ronnie, 2020) [a]		
Just as ICU managers often set the tone for the level of collaboration with agency nurses in their ICUs, so the work relationships between agency and permanent nurses typically followed suit. (Ronnie, 2020)	‘’The permanents shouldn’t look down upon us, like we don’t know anything. Talk to us as human beings. Talk to us, tell us what to do. We do have feelings’’ (Lily, enrolled nurse).	
No matter what the situation, most agreed that a good team required good communication and leadership. (Berg Jansson & Engström, 2017) [a]		
It was suggested that the unit manager’s attitude played a very important role in the agency nurse feeling respected and for the teamwork to be effective. (Muller, 2014)	‘’… for the unit to work to function well it is the leader first to respect the agency staff I have seen this if she is not weighing them at the same level the permanent staff think they are supers of the agency staff irrespective of how senior you are to them I am talking to a level of a assistant nurse and a professional nurse you will see that the assistant nurse is not giving you respect you will see that the assistant nurse don’t respect you as a sister because of the attitude of the unit manger but if the unit manager respect the staff you will see the team work how it goes we will work like sisters and brothers …’’ (Participant 3)	

Synthesis 7: The level of support offered by permanent staff can influence how respected or insecure agency nursing staff feels.

Finding	Direct supporting quotation from the study	Category
Occasionally permanent nurses ignored participants' requests for help, especially if they involved procedures that disrupted particular ward routines. (Manias et al., 2003)	''On this aged care rehab. [rehabilitation] setting I felt that I was disrupting the nurses' routines. Three staff had gone to dinner so that left me with two other nurses. The two other nurses were going around together putting patients to bed. They were not answering any buzzers—I was answering the buzzers. I would say, 'Can I have some help, this patient needs to go to the toilet.' And they would say, 'Oh no, I am putting these patients to bed.' It was just inappropriate.''	
Although the regular CCNs emphasised the necessity to have colleagues to talk with, especially after demanding and ethically challenging situations and mentioned colleagues as a reason to work as regular staff, the agency CCNs did not describe any colleagues as really close to them. (Berg Jansson & Engström, 2017)	''Agency nurses do not always have the help of colleagues, instead they are more alone.'' (CCN 2, agency)	
The lack of response or support leads to feelings of anxiety and vulnerability as mentioned by this participant. (Collier, 2011)	"If you look at a new agency nurse, somebody that comes into a new situation, how vulnerable they feel and the unit is not known, they don't know the staff. Now they don't actually know where they stand and then you find that person is vulnerable because where is the support you want. [...] orientation is very important and I come back to that, it's very important" (Participant 4).	In a new environment, agency nursing staff may feel vulnerable or alone.
The experience of lacking confidence was described through field text as agency nurses' difficulties with multiple workplaces. Inherent in this are the issues of different technology in ICU's and differing approaches to care, both of which contributed to participants' descriptions of insecurity and diminished self-assurance. (Hass et al., 2006) [a]		
… they described feeling as though their skills were being lost and they felt incompetent in having to ask the permanent staff how to undertake simple tasks. (Hass et al., 2006) [a]		

Although some participants enjoyed supportive relationships that served to enrich their practice, others described a lack of supportive consultation especially in settings where they worked regularly and in relatively busy situations. (Manias et al., 2003) [a]		Agency nursing staff experiences varying levels of support from permanent staff.
Participants reported a lack of support from the permanent staff. This lack of support is especially prevalent when the units are very busy and the workload is high despite the usage of agency nurses. During the busy periods, permanent staff would ignore the agency nurses as explained by the following participant: (Collier, 2011)	"There is no support but there is a reason for it, I can't say they do not support but they are so overworked and burnt out, I think that they [permanent staff] got third degree burn out." (Participant 9). "You don't get much support from your colleagues [permanent staff]. You don't have a team spirit" (Participant 8).	
In relation to nursing support, participants believed they received adequate help during their working shift. At times, however, agency nurses did not feel well supported in the clinical area. Interestingly, this situation occurred in settings where they had regularly worked in an environment, and permanent staff perceived that agency nurses were relatively familiar with policies and protocols. (Manias et al., 2003)	Sometimes the person-in-charge has got their own workload and that is where you get problems with support because there is no one around.	
Support from the permanent nurses who have local knowledge can make the nurses more comfortable. [They need] attitudes from the staff that make them feel welcome and safe to ask questions. (FitzGerald et al., 2007)	''… a bit stressful at first but it hasn't been as bad as what I was thinking it would be because the staff are usually helpful and you feel like you can ask if you're not sure.'' (4:202)	Agency nurses felt supported when permanent staff showed respect and shared their knowledge.
On the other hand, different opinions exist in terms of the amount of support that is rendered to agency nurses in the clinical setting. Some participants felt that permanent nurses showed support if the agency nurse had worked most of their shifts in one particular unit. Having worked in one unit for most of their shifts allows for true collaboration by both the agency nurse and the permanent staff. (Collier, 2011)	"There is a lot of support ... they [permanent staff] ask for our inputs, they respect us and they get inputs from us about changes in the system, things like that we give input about our problems, ... the night manager gives us a lot of support and the staff working with us and yes the doctors too" (Participant 10).	
From the interviews conducted, it was apparent that ward nurses were willing to share knowledge with well-trained external staff. (Krebs et al., 2020) [a]		

Synthesis 8: Ever-changing agency nursing staff may pose as a burden on permanent staff.

Finding	Direct supporting quotation from the study	Category
A routine way of working with knowledge of processes, material stock, etc. would thus only develop with a delay and usually in a less well-developed form compared to the internal permanent staff. (Krebs et al., 2020) [a]		Agency nursing staff does not always bring the relief to permanent staff that they are intended for.
In summary, the effectiveness of temporary work was considered to be very low, as external staff did not "know the situation on site that well" and "a lot of money [was] spent on staff who [were] not fully deployable". (Krebs et al., 2020)	"know the situation on site that well"<br>"a lot of money [was] spent on staff who [were] not fully deployable"	
At the same time, it was noted that external staff should be used to relieve the burden, but that this did not occur or was not perceived by the ward staff if the external nurse first had to be shown everything. (Krebs et al., 2020)	"So I think everyone involved has an interest in things running smoothly, but the limits that exist are due to the situation, and if they're only there for two or three days, you can't stand next to them all day."	
In addition, it was clearly pointed out that it is very difficult to integrate external staff into the day-to-day care of a ward. (Krebs et al., 2020) [a]		
[Sufficient induction] was seen as a basis [...] to reduce the "permanent questions" that were perceived as annoying by the permanent staff. (Krebs et al., 2020)	"permanent questions"	Working with constantly new agency nurses can be stressful to permanent staff.
Some of the regular CCNs also mentioned that it sometimes could be draining to constantly meet and introduce new colleagues as a result of an increased level of temporary staffing. The regular CCNs stated that work was more manageable if more regular CCNs or longer term agency CCNs were on duty. (Berg Jansson & Engström, 2017)	''Since we have patients that are really critically ill, then it's reassuring to know that I know what colleagues are with me, and who can do what. It's not that I don't want to be with new CCNs, but you know what it's like, I think it's a bit stressful [to deal with temporary CCNs].'' (CCN 8, regular)	

Synthesis 9: The lack of feedback mechanisms for and from agency nursing staff may hinder professional development and collaboration.

Finding	Direct supporting quotation from the study	Category
Receiving feedback is a method of improving practice. (Hass et al., 2006)	‘‘It is great to get feedback as that is the only way you can improve. If I am better at what I am doing then my patient is going to benefit at the end of the day. It doesn’t matter if it is positive or negative, it is still feedback.’’ (Participant 4)	Agency nursing staff values feedback.
Positive aspects of the agency nurse experience included feedback and support from permanent staff including ICU managers and instances of belonging and acceptance. (Ronnie, 2020) [a]		
Furthermore, fear of reprisal prevents agency nurses to discuss a too heavy workload with the permanent staff. The fear of not being booked to work again is shared by some participants, as at these particular healthcare institutions agency nurses are required on a more regular basis, whilst other healthcare institutions are known for their cancellations. (Collier, 2011)	“Yes, it happens at places that people [agency nurses] are not always communicating. They are scared that they will not be used again” (Participant 4).	A lack of formal feedback mechanisms may hinder the giving and receiving of honest and constructive feedback.
The nurses in the casual pool are clearly in a prime position to assess the ability of clinical teams to provide this type of context [in which agency staff feels safe to function well] in which nurses from the casual pool can work comfortably. (FitzGerald et al., 2007) [a]		
Since agency nurses were not present in the setting for a prolonged period, they all experienced a sense of urgency about voicing any concerns with permanent staff. (Manias et al., 2003)	‘‘In the couple of places where I’ve had really bad experiences, I have told the person-in-charge y If by the end of the shift they haven’t done anything to help me or resolve the issue, I would usually say to them that I was very disappointed in this and for this reason, and that I won’t return to the unit.’’	
The non-existence of receiving honest feedback when working full time as an agency nurse was also discussed. (Hass et al., 2006)	‘‘Feedback really is an essential part, but not getting feedback is just part and parcel of being an agency nurse.’’ (Participant 4)	

<table>
<tr><td>Besides the odd ‘thank you’ at the end of a shift or flattering invitations to ‘come and work with us’, they are not given feedback on their performance. Even complaints about their work are vague. A formal system for enabling casual nurses to provide clinical teams with feedback about their performance in the area would be useful for high performing teams in terms of promoting their area and for poorly performing teams to know where they might start to change practices. [Recommendations made as a result of this study are] A formal feedback mechanism for casual nurses regarding their performance. (FitzGerald et al., 2007)</td><td>‘’… respect really, we don’t get a lot of it, some people on the wards will be [say] thank you for being here, thank you for being able to help us out ...’’ (5:162)</td><td></td></tr>
<tr><td>In addition, the induction period could be used to obtain initial feedback on the mission readiness of these caregivers. (Krebs et al., 2020) [a]</td><td></td><td rowspan="3">Feedback concerning individual agency nurses can be used to exclude them from ward rotation. [a]</td></tr>
<tr><td>However, if there was a feeling that the external personnel had little knowledge and competence, an attempt was made to weed them out. The assessment of the seconded persons took place within a few hours. Either the ward managers received immediate feedback that the ward team did not want to work with a particular external nurse again, or the collaboration developed positively. (Krebs et al., 2020) [a]</td><td></td></tr>
<tr><td>However, aside from refusing to send the agency nurses back to these ward settings, there was no further follow-up with the hospitals. (Manias et al., 2003) [a]</td><td></td></tr>
</table>

Synthesis 10: Agency nursing staff needs to be proactive, confident and self-reliant.

Finding	Direct supporting quotation from the study	Category
Some of the [agency] nurses describe a routine that appears to work for them. They find out more information before starting to look after the patients. (FitzGerald et al., 2007) [a]		Some agency nursing staff has developed an own routine to orient themselves.
On the other days, the new caregiver in question was on her own and had to teach herself everything else by asking specific questions, reading, observing, or the like. (Krebs et al., 2020) [a]		
Participants explained that they often felt as though they were 'disturbing' other staff members when they did not know where to find equipment and dressings. (Hass et al., 2006)	''It is tough on your first day to walk into a new unit and say, can you show me around? to someone who looks like they are really busy ... you know, so sometimes I will avoid that particular stress by arriving half of an hour early and just go and have a look around and sort of orientate myself as to where things are and that way when I start work I have an idea where most things are.'' (Participant 1)	
This reliance upon self-education and individual responsibility appeared to be related to nurses' perceptions about their knowledge deficits. (Manias et al., 2003)	''I think it's your own responsibility to make sure you are on top of things, and if you are not then to find out or access people who can get you up-to-date. Only you would know what your deficits are.''	Agency nursing staff is responsible for filling their own deficits in knowledge.
… One participant commented that when there is inadequate support at the clinical settings, the onus should lie with the agency nurse to request such help. (Collier, 2011)	"I think if I ask for support, it will be there ... and I don't think that we must have that attitude that we are agency-nurses they [permanent staff] must help us all the time. We need to take responsibility and say: I need help now" (Participant 1).	
It is also up to the casual nurse to take an initiative. (FitzGerald et al., 2007)	''go to the ward and only take the first step to mix in and they're usually more than happy to accept you.'' (5:168)	

The participants stressed the need for self-confidence when going to new units and being faced with the possibility of unfamiliar equipment. (Hass et al., 2006) [a]		Confidence and openness are prerequisites for working as an agency nurse.
Likewise, help should be offered [by agency nurses], tasks should be completed in a dedicated and motivated manner, and a friendly approach should be demonstrated to team members as well as to patients on the unit. (Krebs et al., 2020) [a]	‘’certain charisma“<br>‘’certain standing“	
Agency nurses, with a keen sense of their own limitations, had also indicated explicitly to the ICU manager when they felt out of their depth professionally. (Ronnie, 2020)	‘’They allocated me to a very sick patient and I said to the manager ‘’this is outside my scope of practice. I don’t know how to use the machines that they need”. Then they took me off that patient’‘ (Elsie, enrolled nurse).	

ICU: intensive care unit; CCN: critical care nurse.

References

Berg Jansson, A., & Engström, Å. (2017). Working together: critical care nurses experiences of temporary staffing within Swedish health care: a qualitative study. Intensive and Critical Care Nursing, 41, 3–10

Collier, V. (2011). Agency nurses' perceptions of job satisfaction within critical care units in private healthcare institutions. University of Stellenbosch, South Africa: Master's Thesis

FitzGerald, M., McMillan, M., & Maguire, J. M. (2007). Nursing from the casual pool: focus group study to explore the experiences of casual nurses. International Journal of Nursing Practice, 13(4), 229–36

Hass, H., Coyer, F. M., & Theobald, K. A. (2006). The experience of agency nurses working in a London teaching hospital. Intensive and Critical Care Nursing, 22(3), 144–53

Krebs, S., Hasseler, M., & Lietz, A. L. (2020). [Temporary employees in nursing care – rising costs, lack of social and professional acceptance, missing introductory and training programs]. Gesundheitswesen, 82(12), e138-e46

Manias, E., Aitken, R., Peerson, A., Parker, J., & Wong, K. (2003). Agency-nursing work: perceptions and experiences of agency nurses. International Journal of Nursing Studies, 40(3), 269–79

Muller, J. (2014). Experiences of agency nurses regarding their placement in private hospitals in East London, Eastern Cape. University of Fort Hare, South Africa: Doctoral Thesis

Ronnie, L. (2020). Us and them: experiences of agency nurses in intensive care units. Intensive and Critical Care Nursing, 56, 102764
