# Supplementary material for: Induction and Rapid Orientation of Agency Nursing Staff in the Hospital Setting: A Systematic Synthesis of Qualitative Studies
Source: J Adv Nurs. 2025 Feb 27;81(8):5112–29. doi: 10.1111/jan.16840 (PMC12271674; doi:10.1111/jan.16840)
Supplement: Supplementary file 4 — Data S1. [file JAN-81-5112-s004.pdf]

**eMERGE Reporting Guidance**

*France EF, Cunningham M, Ring N, Uny I, Duncan EAS, Jepson RG, et al. Improving reporting of meta-ethnography: the eMERGe reporting guidance. BMC Medical Research Methodology. 2019;19(1):25*

| No. | Criteria | Page |
|---|---|---|
| **Phase 1 - Selecting meta-ethnography and getting started** | | |
| *Introduction* | | |
| 1 | Rationale and context for the meta-ethnography | 2 |
| 2 | Aim(s) of the meta-ethnography | 2-3 |
| 3 | Focus of the meta-ethnography | 2-3 |
| 4 | Rationale for using meta-ethnography | 2 |
| **Phase 2 - Deciding what is relevant** | | |
| *Methods* | | |
| 5 | Search strategy | 4 |
| 6 | Search processes | 4, Supplementary file 1 |
| 7 | Selecting primary studies | 5 |
| *Findings* | | |
| 8 | Outcome of study selection | Figure 1, Supplementary file 1 |
| **Phase 3 - Reading included studies** | | |
| *Methods* | | |
| 9 | Reading and data extraction approach | 5-6 |
| *Findings* | | |
| 10 | Presenting characteristics of included studies | Table 1, Supplementary file 1 |
| **Phase 4 - Determining how studies are related** | | |
| *Methods* | | |
| 11 | Process for determining how studies are related | 5-6 |
| *Findings* | | |
| 12 | Outcome of relating studies | 8, Supplementary file 3 |
| **Phase 5 - Translating studies into one another** | | |
| *Methods* | | |
| 13 | Process of translating studies | 5-6 |
| *Findings* | | |
| 14 | Outcome of translation | Supplementary file 2 |
| **Phase 6 - Synthesizing translations** | | |
| *Methods* | | |
| 15 | Synthesis process | 5-6, Supplementary file 3 |
| *Findings* | | |
| 16 | Outcome of synthesis process | 7-16, Tables 2-4 |
| **Phase 7 - Expressing the synthesis** | | |
| *Discussion* | | |
| 17 | Summary of findings | 16-17 |
| 18 | Strengths, limitations, and reflexivity | 18 |
| 19 | Recommendations and conclusions | 18-19 |
